# Epigallocatechin-3-gallate selenium nanoparticles for neuroprotection by scavenging reactive oxygen species and reducing inflammation

**DOI:** 10.3389/fbioe.2022.989602

**Published:** 2022-09-08

**Authors:** Yiming Wang, Wenqi Luo, Feng Lin, Wanguo Liu, Rui Gu

**Affiliations:** Department of Orthopaedic Surgery, China-Japan Union Hospital of Jilin University, Changchun, China

**Keywords:** spinal cord injury, neuroprotection, epigallocatechin-3-gallate, selenium nanoparticle, reactive oxygen species, inflammation

## Abstract

**Purpose:** Spinal cord injury (SCI) is a severely crippling injury. Scavenging reactive oxygen species (ROS) and suppressing inflammation to ameliorate secondary injury using biomaterials has turned into a promising strategy for SCI recuperation. Herein, epigallocatechin-3-gallate selenium nanoparticles (EGCG-Se NP) that scavenge ROS and attenuate inflammation were used for neuroprotection in SCI.

**Methods:** EGCG-Se NP were arranged using a simple redox framework. The size, morphology, and chemical structure of the EGCG-Se NP were characterized. The protective effect of EGCG-Se NP for neuroprotection was examined in cell culture and in an SCI rat model.

**Results:** EGCG-Se NP could promptly scavenge excess ROS and safeguard PC12 cells against H_2_O_2_-induced oxidative harm *in vitro*. After intravenous delivery in SCI rats, EGCG-Se NP significantly improved locomotor capacity and diminished the injury region by safeguarding neurons and myelin sheaths. Component studies showed that the main restorative impact of EGCG-Se NP was due to their ROS-scavenging and anti-inflammatory properties.

**Conclusion:** This study showed the superior neuroprotective effect of EGCG-Se NP through ROS sequestration and anti-inflammatory capabilities. EGCG-Se NP could be a promising and effective treatment for SCI.

## Introduction

Spinal cord injury (SCI) can cause irreversible neurological deficits and can affect the bladder, gut, and sexual capacity, bringing about a critical decrease to quality of life, SCI is commonly induced by trauma or sudden external force, termed traumatic SCI, and is typically occurring in car accidents, high falls, sports and other accidents (Ahuja et al., 2017b; Ropper and Ropper, 2017; Zrzavy et al., 2021). SCI includes primary injury and downstream harmful effects, referred to as “secondary injury,” which exasperates the underlying effect and causes neighboring neuron death (Andrabi et al., 2020). A significant feature of secondary injury is that excess reactive oxygen species (ROS) are created in the damaged spinal cord (Kim et al., 2017; Song et al., 2019). In addition, overproduction of ROS can cause signiﬁcant oxidative harm to biomolecules, including lipids, proteins, and DNA, which not only increases the infiltration of macrophages and neutrophils into the area but also activates microglia in the injured spinal cord (Ahuja et al., 2017b). These immune cells release inflammatory cytokines, like TNF-α and IL-6, in addition to ROS, thereby enhancing cell invasion into the injured spinal cord and increasing inflammation (Ahuja et al., 2017a; Desai et al., 2017; Kim et al., 2017). ROS overproduction is considered a pivotal part of the cascade of secondary injury in SCI (Kim et al., 2017; Li et al., 2019; Zhang et al., 2021). Therefore, scavenging ROS and suppressing inflammation to improve the microenvironment after injury is vital for SCI treatment (Xiao et al., 2020; Yang et al., 2020; Zhang et al., 2020; Zrzavy et al., 2021).

To this end, different kinds of antioxidative materials had been investigated for their neuroprotective properties in preclinical SCI models (Li et al., 2019; Luo et al., 2020; Zhang et al., 2021; Cui et al., 2022; Liu et al., 2022). Zhang et al. showed that lipid-polymer nanoparticles (NP) with high ROS-scavenging capacity alleviated long-haul secondary injury in a clinically applicable rodent SCI model (Zhang et al., 2021). Li et al. showed that tetramethyl pyrazine-stacked NP had significant antioxidant and anti-inflammatory properties which could forestall secondary injury and improve locomotor abilities (Li et al., 2021). In addition, release of antioxidant enzymes, which are effective ROS foragers, to the site of injury could alleviate SCI-induced oxidative pressure and tissue damage (Nukolova et al., 2018; Andrabi et al., 2020). Researchers have indicated that infusion of cerium oxide NP into the injured spinal cord of rats could decrease ROS levels, lessen irritation and apoptosis, and improve locomotor practical recovery (Kim et al., 2017). The ROS-scavenging nitroxide radical 2,2,6,6-tetramethylpiperidin-1-oxyl (TEMPO) has been attached to the side chains of polymers to control ROS levels. Zhang et al. integrated a hydrogel with TEMPO and hyaluronic acid, which furthered neuroprotection and recuperation for SCI (Zhang et al., 2020).

Selenium (Se) is a minor but significant element in human health (Rao et al., 2019). The main function of Se is through glutathione peroxidase (GPX)-associated defense against oxidative stress (Bai et al., 2017; Cong et al., 2019). Moreover, studies have recognized that Se has neuroprotective impacts in the central nervous system, with critical advantages against neurodegenerative diseases (Zhai et al., 2017; Cong et al., 2019; Rao et al., 2019). Previous investigations have also demonstrated that Se could prevent secondary pathological events in severe cases of SCI and reduce functional deficits *via* its antioxidant properties (Li et al., 2011; Zhai et al., 2017; Heller et al., 2019). Se NP has excellent antioxidant properties, low toxicity, and exceptional biocompatibility and degradability (Li et al., 2011; Zhai et al., 2017; Cong et al., 2019).

**FIGURE 1:**
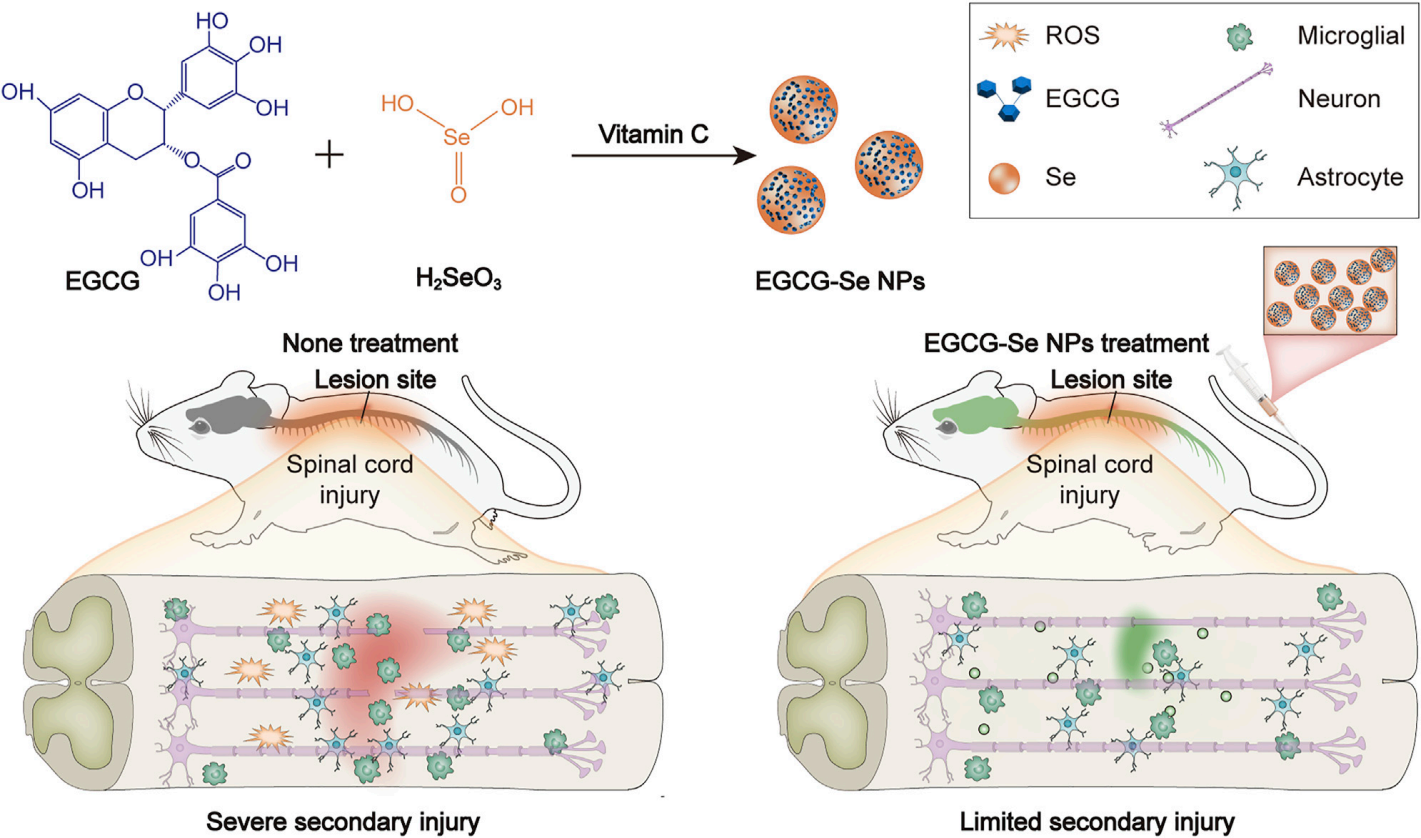
Preparation of epigallocatechin-3-gallate selenium nanoparticles (EGCG-Se NP) for treatment of spinal cord injury by scavenging reactive oxygen species (ROS) and suppressing inflammation.

In the current study, Selenium is an essential component of the antioxidant system *in vivo*. The selenium-doped carbon quantum dots (Se-CQDs) constructed in our previous work exhibited efficacy in protecting cells from lipid peroxidation damage and inflammation regulation in an animal model of SCI (Luo et al., 2020). In light of the above-mentioned work, the present study encapsulated selenium nanoparticles by epigallocatechin-3-gallate (EGCG), which not only endowed the nanoparticles with excellent solubility and biocompatibility, but also possessed more potent antioxidant and anti-inflammatory properties, opening up a new avenue for SCI therapy (Chen et al., 2021; Lee et al., 2021; Ma et al., 2021). EGCG -Se NPs were prepared to ameliorate secondary injury by scavenging ROS and suppressing inflammation in the injured spinal cord (Figure 1). EGCG-Se NP were integrated through a simple redox response using EGCG as a stabilizer and capping agent as already reported (Zhang et al., 2010). EGCG-Se NP had great biocompatibility and exceptional ROS-scavenging properties. *In vivo* experiments, treatment with EGCG-Se NP showed promising effects on neurological capacity in terms of improvement of secondary injury, altogether presenting an effective agent for the treatment of SCI and possibly other ROS-related diseases.

## Materials and methods

### Materials

EGCG and selenious acid were obtained from Shanghai Macklin Biochemical (Shanghai, China), 3-(4,5-dimethyl-thiazol-2-yl)-2,5-diphenyl tetrazolium bromide (MTT) and 4′,6-diamidino-2-phenylindole dihydrochloride (DAPI) were obtained from Solarbio Science & Technology (Beijing, China), sodium ascorbate was obtained from Aladdin Bio-Chem Technology (Shanghai, China), and Dulbecco’s modified Eagle’s medium (DMEM) and fetal bovine serum were obtained from Thermo Fisher Scientific (Waltham, MA). Anti-Iba-1, anti-caspase 3, anti-NeuN, anti-GFAP, and anti-GPX 1 antibody were bought from Sigma-Aldrich (St. Louis, MO). Anti-CD68, anti-NF200, and anti-superoxide dismutase (SOD) antibodies were bought from Abcam (Cambridge, United Kingdom). Hydrogen peroxide (H_2_O_2_, 30 wt% in water) was bought from Shanghai Macklin Biochemical.

### EGCG-Se NP preparation and characterization

EGCG-Se NP were prepared as previously described (Zhang et al., 2010). Briefly, 2.75 g of EGCG (6 mmol) and 0.129 g of selenious acid (1 mmol) were added to 4 ml of 75% ethanol aqueous solution and stirred to completely dissolve. Then, 10 ml of sodium ascorbate solution (5 M) were dripped into the mixture, and the reaction took place overnight in a nitrogen atmosphere. After dialysis and lyophilization, EGCG-Se NP was obtained.

The size distribution of EGCG-Se NP was assessed with a ZEN3600 instrument (Malvern, Worcestershire, United Kingdom). Transmission electron microscopy (TEM) images were obtained with a JEM-1011 microscope (JEOL, Tokyo, Japan) at an accelerating voltage of 100 kV. X-ray photoelectron spectroscopy was performed with an X-ray surface photoelectron spectrometer (Thermo ESCALAB 250, United Kingdom). The Fourier transform infrared spectra (FT-IR) and ultraviolet-visible absorption spectra were acquired utilizing a Win-IR spectrometer (Bio-Rad, Hercules, CA) and a UV-Lambda365 spectrophotometer (PerkinElmer, Waltham, MA), respectively.

### *In Vitro* antioxidant effects of EGCG-Se NP

The test configuration was listed below: control group: water (2 ml), diphenylpicrylhydrazyl (DPPH) anhydrous ethanol solution (0.4 mM, 2 ml); experimental group: EGCG-Se NP (1.25–20 μg/ml, 2 ml), DPPH anhydrous ethanol solution (0.4 mm, 2 ml); blank group: EGCG-Se NP (1.25–20 μg/ml, 2 ml), absolute ethanol (2 ml). The background was adjusted with a combination of water (2 ml) and anhydrous ethanol (2 ml). All groups were cultured in the dark for 30 min and the absorbance at 517 nm was measured with a Bio-Rad 680 microplate reader. The free radical-scavenging rate (%) was calculated by this equation: scavenging rate (%) = (1 − (absorbance value of the experimental group − absorbance value of the blank group)/absorbance value of the control) × 100.

### Cytotoxicity and protection from H_2_O_2_-Induced oxidative stress *in vitro*

PC12 cells were obtained from the Cell Bank of the Chinese Academy of Sciences (Shanghai, China), and BV2 microglia were purchased from BNCC (Beijing, China). The cytotoxicity of EGCG-Se NP in PC12 cells was assessed via an MTT test. Briefly, PC12 cells were cultured in 96-well plates at 7,000 cells/well and incubated overnight. Then, the medium was aspirated and 200 μL of new medium containing EGCG-Se NP with various concentrations were added. After incubating for 24 or 48 h, the MTT test was performed following the standard method. The absorbance of all was measured at 490 nm.

To investigate the protective properties of EGCG-Se NP in H_2_O_2_-induced oxidative stress, PC12 cells were cultured in 96-well plates at 7,000 cells/well in 180 μL of DMEM and incubated for 24 h. The media was pretreated with indicated concentrations (0–100 μg/ml) of EGCG-Se NP or phosphate-buffered saline (PBS) for 30 min. Then, the media was incubated with 500 μm H_2_O_2_ for 24 h. Cell viability was measured with the MTT test using a Bio-Rad 680 microplate reader and live/dead cell staining using a confocal laser scanning microscope (LSM780; Carl Zeiss Meditec, Jena, Germany). The live cell numbers were determined with ImageJ (National Institutes of Health, Bethesda, MD). The levels of ROS in PC12 cells incubated with EGCG-Se NP were quantified by estimating the fluorescence intensity with 2′,7′-dichlorofluorescein diacetate (DCFH-DA; Sigma-Aldrich) *via* confocal laser scanning microscopy.

### *In Vitro* anti-inflammatory effects of EGCG-Se NP

The anti-inflammatory ability of EGCG-Se NP was assessed by an enzyme-linked immunosorbent assay (ELISA). BV2 cells were seeded in 6-well plates at a density of 1.5 × 10^5^ cells per well and cultured for 24 h. After that, lipopolysaccharide (LPS) was added to stimulate cells for 30 min. Then, an equal volume of PBS or EGCG-Se NP were added to the cell culture medium (final concentration of NP, 10 μg/ml). After 24 h, the supernatant was collected and pro-inflammatory cytokines were measured by ELISA.

### Rat model of SCI

Adult Sprague-Dawley rats (inbred strain, female, 200–250 g) were bought from Liaoning Changsheng Biotechnology Co., Ltd. Rats were housed with sufficient water and food in a 12-h/12-h light-dark cycle environment and under controlled temperature (23 ± 2°C). The Animal Ethics Committee of Jilin University approved the animal protocols (No. SY202103013). We used the weight-drop SCI model (Lin et al., 2022). In simple terms, rats were deeply anaesthetized with pentobarbitone sodium, and thoracic laminectomy was performed at the T10 level. The rats received a moderate contusion injury (40 g weight, 50 mm height) to expose the spinal cord at T10 using an impactor. Following a medical procedure, the rats were returned to their enclosures with plenty of water and food. Intraperitoneal injections of sodium ampicillin (80 mg/kg) were performed for 5 days (Zhang et al., 2021). The bladder was squeezed two times per day until there was no more than 0.5 ml urine per day.

### Locomotor function assessment

The rats were grouped as follows: a control group without any surgery, and five groups treated with either saline, methylprednisolone sodium succinate (MP), 9.5 mg/kg EGCG, 10 mg/kg EGCG-Se (EGCG-Se high-dose group, EGCG-Se H), or 5 mg/kg EGCG-Se (EGCG-Se low-dose group, EGCG-Se L) after surgery. The restoration of hind limb motor function was assessed by the Basso, Beattie, and Bresnahan (BBB) motor rating score. The scores were finalized when both independent researchers who conducted the tests agreed. The recovery time of postoperative urinary function was also recorded.

### Histological analysis and immunofluorescence

At a predetermined time, the rats were sacrificed by excessive anesthesia and perfused with 0.9% saline followed by 4% paraformaldehyde. Spinal cord tissue (2 cm fragments) and other organs were collected, embedded in paraffin, and sectioned coronally. Hematoxylin-eosin (H&E) and Luxol fast blue (LFB, 0.1%) staining were performed, and then sections were imaged under a Panoramic 1,000 optical microscope (3DHISTECH, Budapest, Hungary). The spinal cord was then dissected, cut into 1-mm^3^ sheets, post-immobilized overnight in 2.5% glutaraldehyde at 4°C, transferred to osmication (90 min) and dehydration (140 min), and then uranyl acetate and lead citrate TEM (HT7700, Hitachi) was used to evaluate the restorative effect on demyelination. The number of myelin sheaths was evaluated using ImageJ. Immunofluorescence staining assays were also conducted as follows. The primary antibodies (NeuN, 1:500; NF200, 1:200; GFAP, 1:1,000; Iba-1, 1:200; CD68, 1:200; caspase 3, 1:1,000; GPX 1, 1:200; SOD, 1:200) were blocked (3% bovine serum albumin) for 1 h and then incubated overnight at 4°C. The sections were washed 3 times, then incubated in the dark with the secondary antibody for 1 h at room temperature before labeling the nucleus with DAPI. Observation of immunofluorescence sections was conducted by confocal laser scanning microscopy.

### Statistical analysis

All numeric data were presented as mean ± standard deviation. Repeated-measures one-way analysis of variance or t-tests were used in GraphPad Prism (version 8.0.2; GraphPad Software, San Diego, CA). Significance was determined at *p* < 0.05.

## Results and discussion

### Preparation and characterization of EGCG-Se NP

**FIGURE 2:**
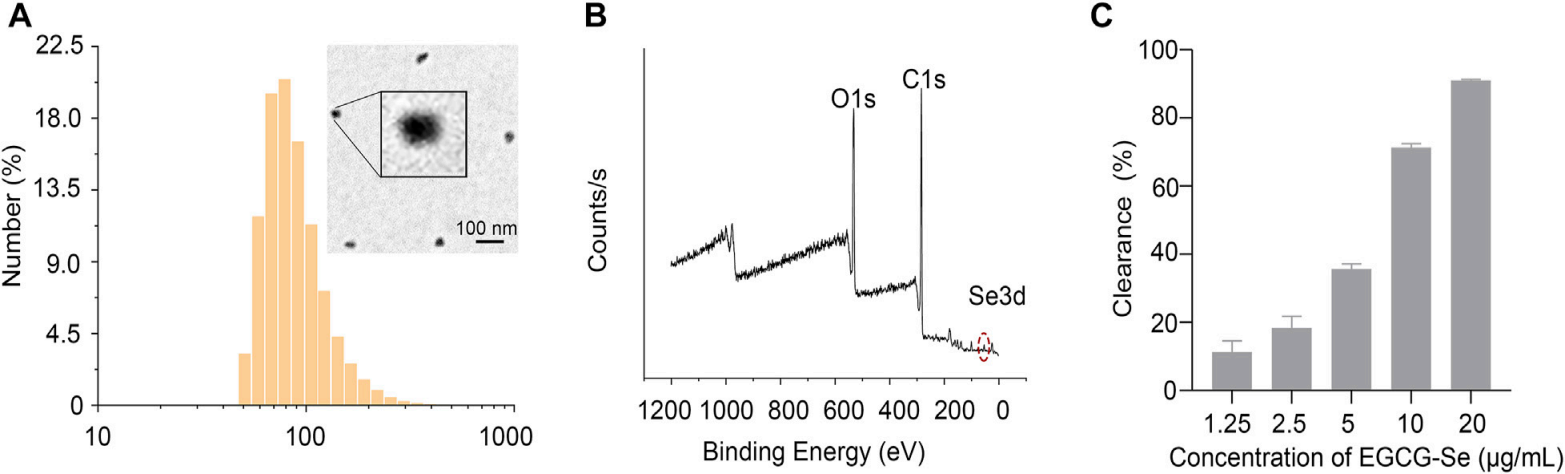
Characterization of epigallocatechin-3-gallate selenium nanoparticles (EGCG-Se NP). **(A)** EGCG-Se NP size distribution was detected by dynamic laser scattering and morphological image of an EGCG-Se NP was obtained via transmission electron microscopy. Scale bar = 100 nm. **(B)** X-ray photoelectron spectroscopy spectrum of EGCG-Se NP. **(C)** Clearance of diphenyl picrylhydrazyl by EGCG-Se NP.

The experimental design is described in Figure 1. First, EGCG-Se NP were prepared as per previously described (Zhang et al., 2010). The hydrodynamic particle size of the EGCG-Se NP was 91.3 ± 35.7 nm. Morphological TEM images of EGCG-Se NP show particles with a rounded shape, confirming their successful synthesis (Figure 2A). Moreover, the typical Se 3 d peak of Se (0) at 55 eV and the O1s peak of the hydroxyl group of EGCG were detected at 532 eV, indicating the successful combination of EGCG and Se (Figure 2B). According to the results of ICP-MS, the selenium content in EGCG-Se NPs is about 4.7%. FT-IR was used to further confirm this combination of EGCG and Se; the stretching vibration peaks of −OH, O=C−O, and C−O were displayed at 3,360, 1,620, and 1,150 cm^−1^, which further confirmed that EGCG was combined with Se (Supplementary Figure S1) (Huang et al., 2019). In addition, the characteristic peak of the −OH group in EGCG alone at 3,360 cm^−1^ is higher relative to that of EGCG-Se NPs, indicating that EGCG is conjugated to the Se surface through this functional group. Together, these results indicate the successful synthesis of EGCG-Se NP. Given the inconvenient impacts of abundance ROS (Wiegman et al., 2015; Choi et al., 2019; Cong et al., 2019; Ashrafizadeh et al., 2020; Rosenkrans et al., 2020), we evaluated the ROS-scavenging capability of EGCG-Se NP *via* the DPPH test. As shown in Figure 2C and Supplementary Figure S2, EGCG-Se NP effectively removed ROS in a dose-dependent manner, thus demonstrating their potential to scavenge ROS. The effective scavenging ability of EGCG-Se NP is attributed to the oxidation of phenolic hydroxyl groups in the NP induced by ROS (Lee et al., 2021). This ROS scavenging ability enables EGCG-Se NP to protect cells against oxidative damages, which may inhibit the inflammatory response and mitigate the secondary injury in spinal cord injury.

### Biocompatibility and antioxidant effect of EGCG-Se NP

**FIGURE 3:**
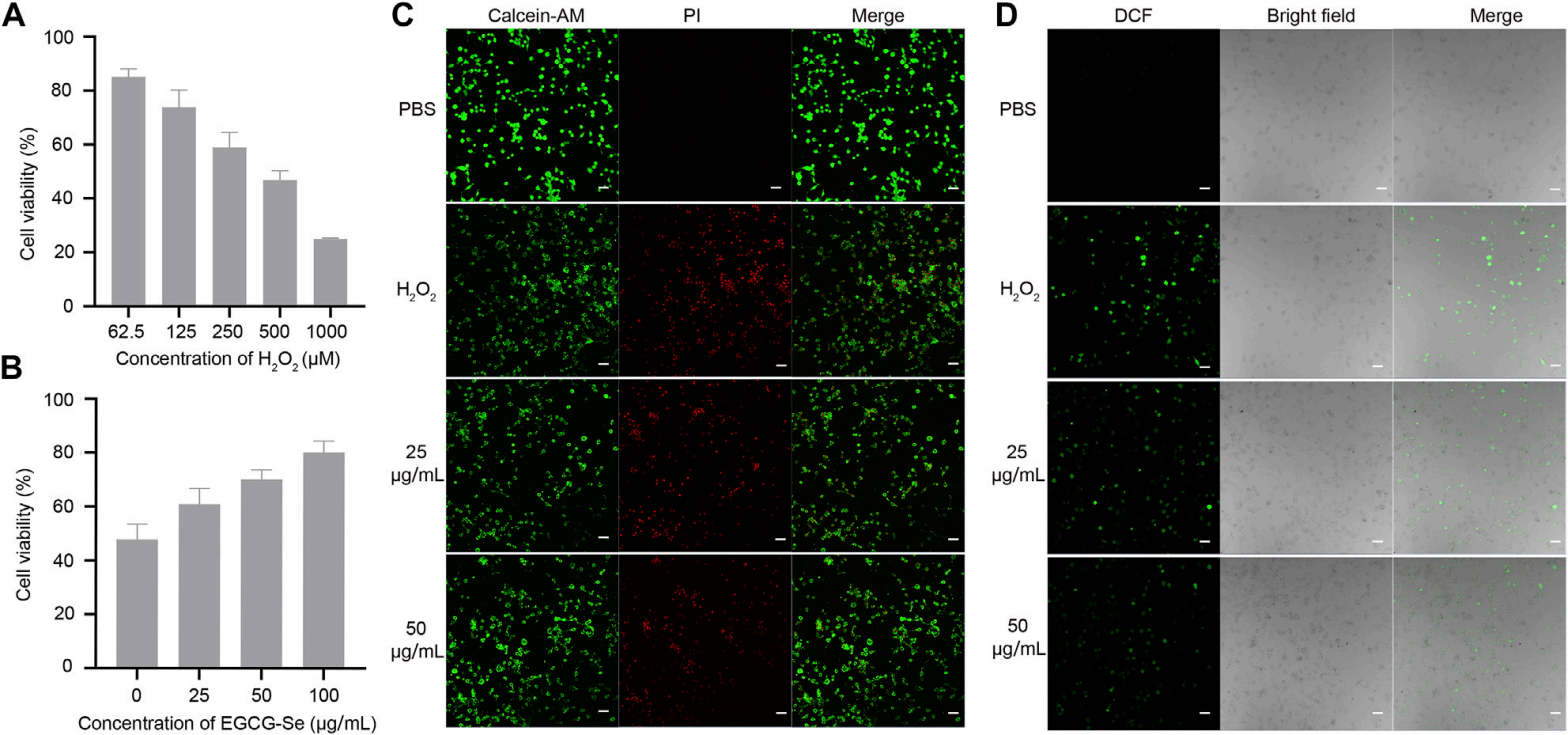
Epigallocatechin-3-gallate selenium nanoparticles (EGCG-Se NP) can protect PC12 cells from H_2_O_2_-induced damage. **(A)** H_2_O_2_-induced damage effect of PC12 cells. **(B)** EGCG-Se NP increased PC12 cell viability under 500 μm H_2_O_2_-induced oxidative damage in a dose-dependent manner. **(C)** Live/dead staining of PC12 cells under different conditions. Scale bar = 20 μm. **(D)** Dichlorofluorescein (DCF) diacetate staining to detect intracellular reactive oxygen species in PC12 cells. PBS, phosphate-buffered saline; PI, propidium iodide.

To assess the biocompatibility of EGCG-Se NP, cytotoxicity tests were performed. PC12 cell viability was assessed after exposure to different concentrations of EGCG-Se NP, which exhibited no observable cytotoxicity up to a concentration of 100 μg/ml (Supplementary Figure S3). To mimic oxidative damage of cells induced by ROS, different concentrations of H_2_O_2_ were added to the medium and the number of dead PC12 cells were measured for each (Figure 3A). As shown in Figure 3B, EGCG-Se NP prevented PC12 cell death in a dose-dependent manner. The protective ability of EGCG-Se NP against H_2_O_2_-induced cell death was further affirmed by live/dead cell staining, whereby the number of viable cells significantly increased upon treatment with EGCG-Se NP (Figure 3C; Supplementary Figure S4), consistent with the cytotoxicity tests. Next, intracellular ROS levels were estimated in different treatments. As shown in Figure 3D; Supplementary Figure S5, the fluorescence intensity of dichlorofluorescein diacetate (DCF) significantly decreased with EGCG-Se NP treatment. This indicated that the ROS levels were effectively reduced by EGCG-Se NP, consistent with the results in Figures 2C, 3B. These results confirmed that EGCG-Se NP can protect PC12 cells from H_2_O_2_-induced damage by effectively scavenging ROS. Furthermore, LPS-activated microglia were tested to evaluate the anti-inflammatory ability of EGCG-Se NP. TNF-α and IL-6 levels were downregulated after treatment with EGCG-Se NP compared to the LPS-treated groups (Supplementary Figure S6). The data indicated that EGCG-Se NP readily reduced the inflammatory microglial response by scavenging ROS (Kim et al., 2017).

### Functional recovery of SCI rats following treatment

**FIGURE 4:**
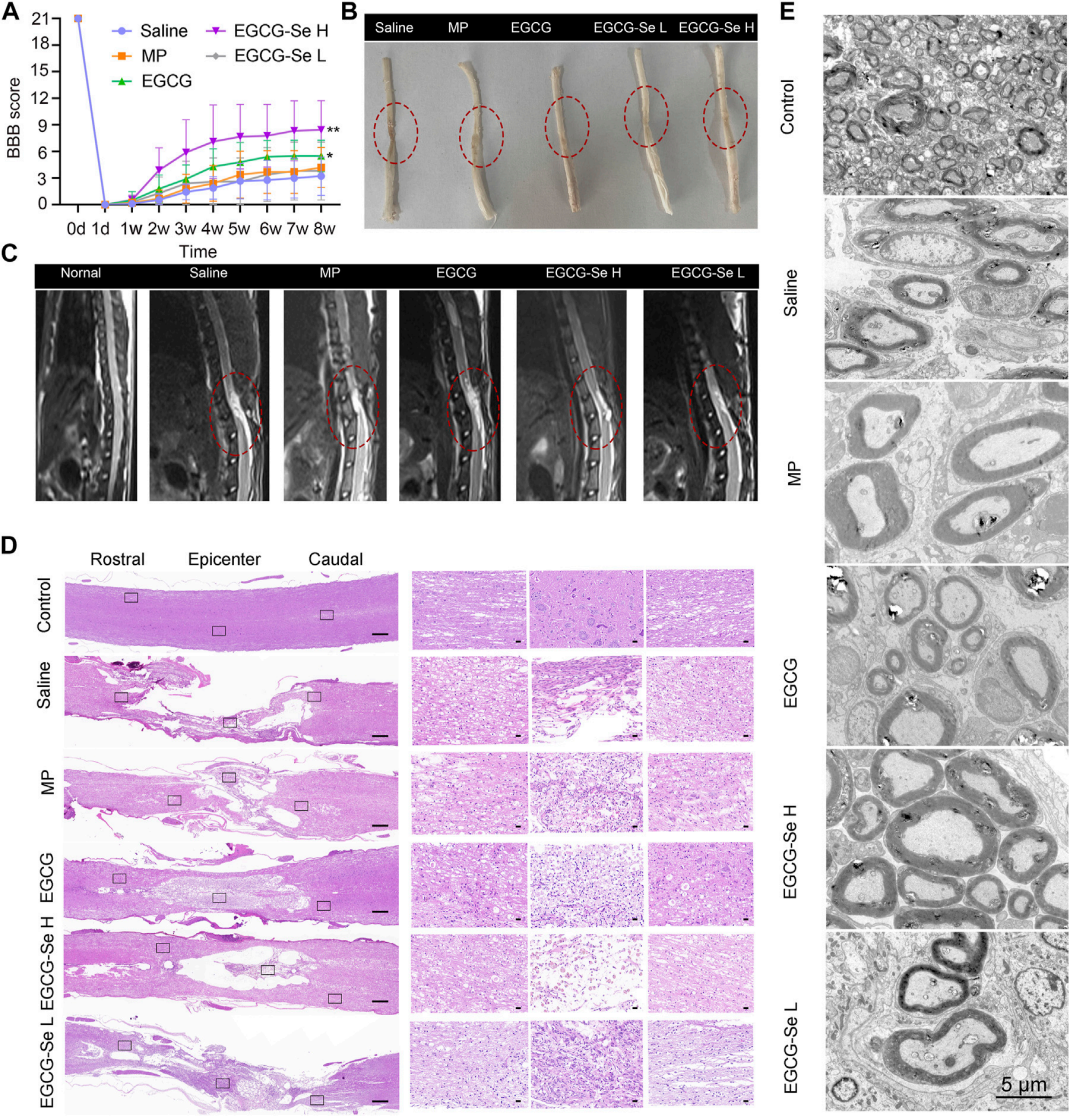
Assessment on the recovery of motor function after spinal cord injury (SCI). **(A)** Basso, Beattie, and Bresnahan (BBB) scores of SCI rats treated with saline, methylprednisolone sodium succinate, 9.5 mg/kg epigallocatechin-3-gallate (EGCG), 5 mg/kg EGCG-Se nanoparticles (NP), or 10 mg/kg EGCG-Se NP. **p < 0.01, *p < 0.05, in comparison with the saline group. **(B)** Images of the spinal cord in different treatment groups 8 weeks after SCI. The lesion of spinal cord injury is indicated in the red dashed circle. **(C)** T2-weighted sagittal magnetic resonance images of the spinal cord following different treatments, in which the red oval represents the injury site. **(D)** Representative hematoxylin-eosin staining images of spinal cord tissue. The black rectangle on the left shows the small image on the right after zooming in. Scale bars are 500 μm (left) and 50 μm (right), respectively. **(E)** Representative transmission electron microscopic images of myelin sheath ultrastructure. Scale bar = 5 μm.

Considering that EGCG-Se NP can scavenge ROS and suppress inflammation *in vitro*, we examined the neuroprotective effect of EGCG-Se NP after acute SCI in a T10 contusion rat model. A locomotor function study was conducted to determine whether EGCG-Se NP could improve lower extremity motor function in injured animals. First, five or 10 mg/kg EGCG-Se NP, 0.9% saline, 9.5 mg/kg EGCG, or 30 mg/kg MP were given intravenously within 5 min after injury. According to the previous study and the ICP-MS results of EGCE-Se NP, we chose the concentration of EGCG-Se NP and EGCG was 10 mg/kg, 9.5 mg/kg, respectively (Cheng et al., 2021). The therapeutic concentration for MP is the clinically recommended therapeutic concentration for spinal cord injury (Ahuja et al., 2017b; Zhang et al., 2018). MP is one of most frequently used drug for clinical treatment of SCI, which has been routinely established as a control group in animal studies (Huang et al., 2020; Lin et al., 2022). The BBB score of rats treated with 10 mg/kg EGCG-Se NP (8.4 ± 0.5) was significantly better than that of the saline group (3.2 ± 0.4) at 8 weeks after surgery (Figure 4A). Moreover, the therapeutic effect of 10 mg/kg EGCG-Se NP was better than that of 5 mg/kg. While rats treated with saline had sweeping hind limbs without weight support, rats treated with EGCG-Se NP could frequently support their weight on their feet and occasionally showed hindlimb coordination with forelimbs (Supplementary Figure S7). In addition, the area of SCI in rats treated with EGCG-Se NP was smaller than that in rats treated with saline (Figure 4B). To comprehensively provide evidence of lower extremity motor function recovery, 3-T magnetic resonance imaging (MRI) was conducted to evaluate the spinal cord changes in each group. T2-weighted MRI demonstrated high signal intensity at the site of injury, presenting as a fluid-filled cyst. Compared with the saline-treated group, rats treated with EGCG-Se NP had better treatment effect, and a more intact spinal cord and smaller cyst volume were observed in the EGCG-Se NP group (Figure 3C). This is in accord with previous studies (Wang et al., 2019a; Wang et al., 2019b).

To understand the anatomical changes involved in the lower extremity motor function recovery, histomorphological changes of the spinal cord were detected by H&E staining at 8 weeks after injury. The integrality, as well as the consecutiveness of the spinal cord structure presented the worst in rats treated with saline, and glial proliferation was observed around the large cystic space (Figure 4D). In contrast, the lesion area and lumen volume were reduced in rats treated with 5 mg/kg EGCG-Se NP and MP, while the reduction was more pronounced in the 10 mg/kg EGCG-Se NP group (Figure 4D). Overall, EGCG and MP treatment significantly improved spinal cord continuity and reduced lesion voiding, with 10 mg/kg EGCG-Se NP having the most significant protective effect. SCI often results in severe axonal demyelination and myelin structure injury (Ramer et al., 2014; Silva et al., 2014; Cunha et al., 2020; Floriddia et al., 2020). Next, we used LFB staining and TEM to evaluate the changes of demyelination and myelin sheath ultrastructure at the lesion site (Supplementary Figure S8; Figure 4E). Compared with the control group, the saline group showed obvious demyelination with damage to the myelin sheath ultrastructure at 8 weeks after injury. Rats treated with EGCG-Se NP had more myelin sheath and more intact ultrastructure in a dose-dependent manner. Compared with the control group, the amount of myelin sheath in the saline group was significantly reduced, while it was significantly increased in the EGCG-Se NP group (*p* < 0.001) (Supplementary Figure S9). In general, the LFB staining and TEM showed that EGCG-Se NP treatment had beneficial effects on demyelination and preservation of nerve fibers in SCI models.

**FIGURE 5:**
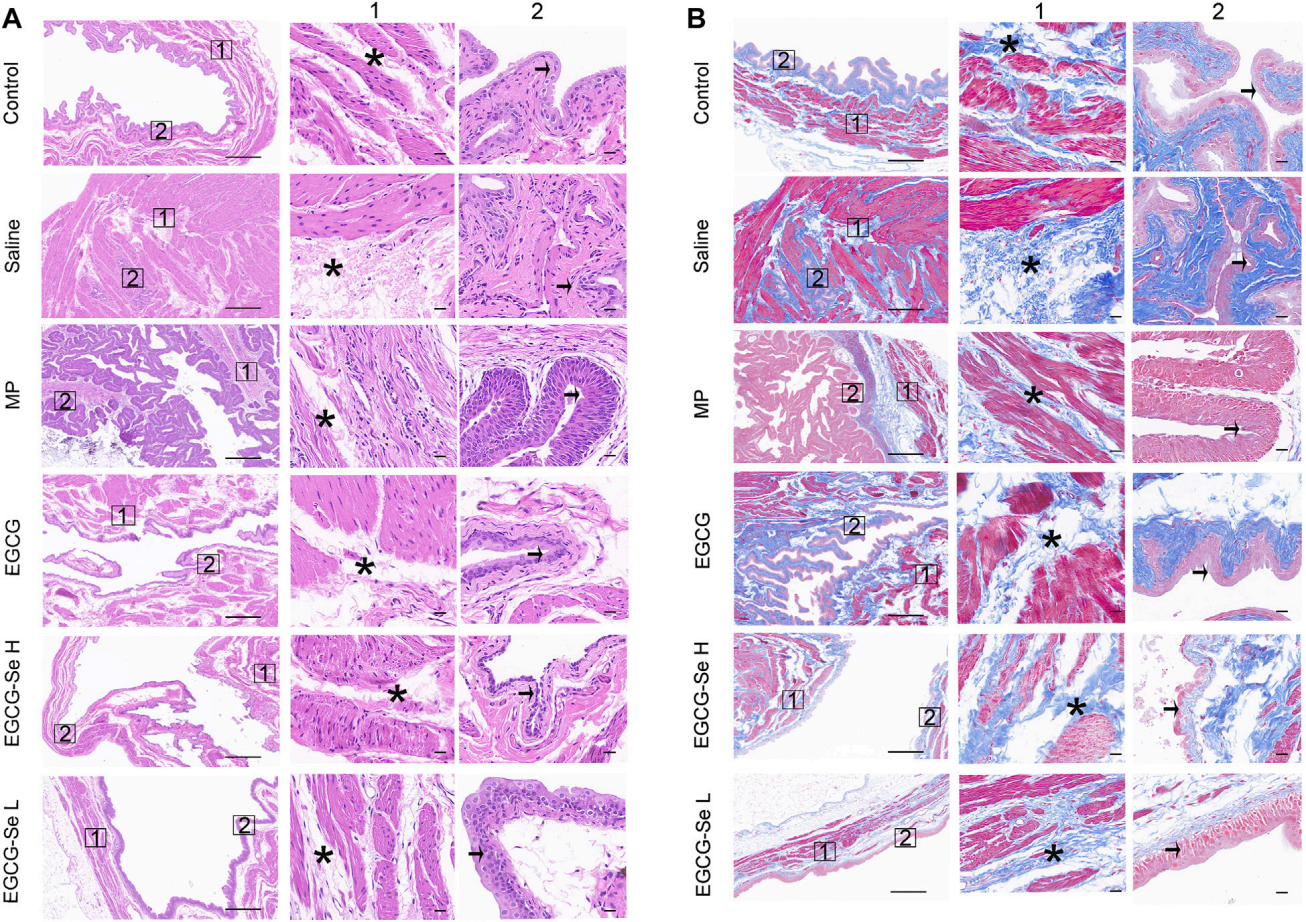
Hematoxylin-eosin (H and E) and Masson staining of bladders. **(A)** Representative bladders stained with H&E. Columns 1 and 2 are magnifications of the areas marked by the blue squares in the left column. Pathological changes of the bladder are indicated using asterisks (muscle layer) and arrows (intima). Scale bars are 500 and 50 μm, respectively. **(B)** Representative bladders stained with Masson stain. Columns 1 and 2 are magnifications of the areas marked by the blue squares in the left column. Pathological changes of the bladder are indicated using asterisks (muscle layer) and arrows (intima). Scale bars are 500 and 50 μm, respectively.

Neurogenic bladder is a complication of SCI the main symptoms were incomplete bladder emptying, chronic urinary retention, and increased bladder pressure lead to renal failure and the bladder will show pathological changes of bladder wall fibrosis and bladder endometriosis (Hamid et al., 2018; Li et al., 2019; Luo et al., 2020). We hypothesized that EGCG-Se NP therapy may benefit bladder tissue protection by reducing secondary injury. As shown in Supplementary Figure S10, the recovery rate of natural urination was faster in rats treated with 10 mg/kg EGCG-Se NP than in those treated with saline. Bladder tissues were stained with H&E and Masson staining to further assess bladder function recovery. In contrast to the saline group, the EGCG-Se NP group revealed significantly limited pathological damage to bladder tissue and reduced levels of bladder wall fibrosis and bladder endometriosis (Figures 5A,B). This may be due to the promoting of neurological recovery and early restoration of spontaneous urination by EGCG-Se NP.SCI results in muscle atrophy of the lower limbs due to a lack of neurotrophic factors (Sangari et al., 2019; Kutschenko et al., 2022). To detect the amyotrophic effect of EGCG-Se NP on SCI in rats, H&E staining, the muscle/weight ratio, and Masson staining of the gastrocnemius muscle were evaluated. As shown in Supplementary Figures S11, S12, no significant differences were observed across these groups. The reason may be that the significant lower limb muscle atrophy caused by spinal cord injury occurs 8 weeks after the injury, or even longer. Therefore, the results of this experiment are negative (Koh et al., 2017).

### Immunofluorescent characterization of SCI rats after EGCG-Se NP treatment

**FIGURE 6:**
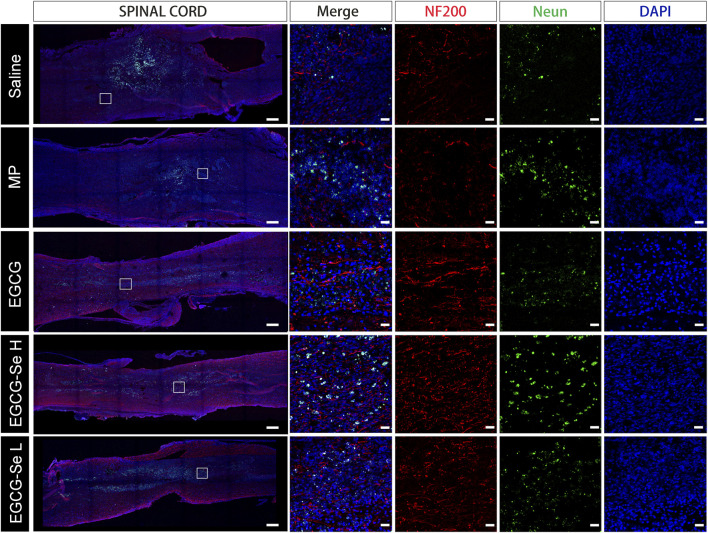
Anti-NF200 and anti-NeuN double-staining of different groups at 8 weeks post-injury. Scale bars are 400 μm in the first column and 25 μm in the other columns. MP, methylprednisolone; EGCG, epigallocatechin-3-gallate; L, 5 mg/kg EGCG-Se nanoparticles; H, 10 mg/kg EGCG-Se nanoparticles.

**FIGURE 7:**
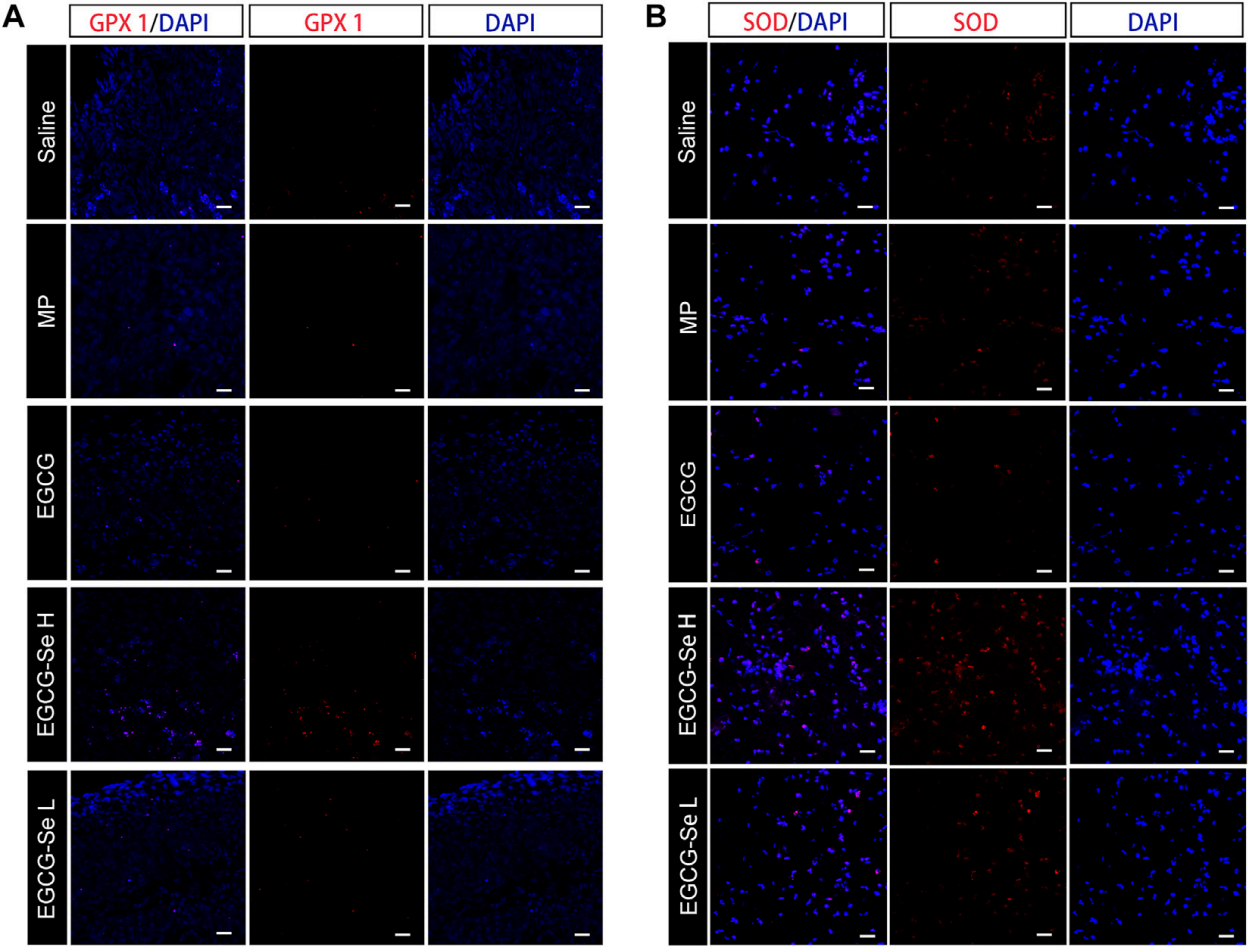
Representative immunohistochemical staining of antioxidant enzymes in the injured spinal cord. **(A)** GPX one staining at 8 weeks post-injury. **(B)** SOD staining at 1-day post-injury. Scale bars 25 μm.

To detect the neuroprotective effect of EGCG-Se NP in SCI in rats, anti-NF200 and anti-NeuN immunofluorescent staining were performed. Compared with rats treated with 10 mg/kg EGCG-Se NP, the expression levels of NF200 and NeuN were significantly decreased in the saline group (Figure 6), NF200 is a neurofilament protein that provides structural support for axons and regulates axon diameter. NeuN is common in mature neurons. Low levels of expression indicate poor spinal cord recovery, suggesting that high concentrations of EGCG-Se NP have a neuroprotective effect, thus promoting the recovery of spinal cord function in treated animals. Previous studies have reported that scavenging ROS can inhibit the inflammatory response of SCI secondary injury (Ren et al., 2018; Zheng et al., 2019; Bai et al., 2020; Xiao et al., 2020). Thus, we investigated the inflammation suppression activity of EGCG-Se NP on days 1 and 56 *in vivo.* Immunofluorescent staining showed an increase in the number of CD68-positive cells at the injury site in saline-treated rats compared with the control group. By comparison, there were fewer CD68-positive cells in the spinal cords of rats treated with EGCG-Se NP compared with saline (Supplementary Figures S13, S15), suggesting that high concentrations of EGCG-Se NP were effective in reducing inflammation at the site of injury. Studies have shown that eliminating ROS at the site of injury by antioxidant therapy is an effective neuroprotective strategy after acute SCI (Tyler et al., 2013; Wu et al., 2014; Krupa et al., 2019; Zhang et al., 2021). Therefore, to further understand the potential mechanism of the therapeutic effect of EGCG-Se NP, we measured the levels of antioxidant enzymes in rats treated with EGCG-Se NP 1 day and 56 days post-injury. Compared with the saline group, the expression levels of GPX one and SOD in rats treated with 10 mg/kg EGCG-Se NP were significantly increased (Figure 7), indicating that high concentrations of EGCG-Se NP could promote antioxidant enzymes and reduce ROS levels (Cong et al., 2019). The long-term neurological deficits after SCI are partly due to the extensive activation of neurons and oligodendrocyte apoptosis at the injury site (Springer et al., 1999; Ahuja et al., 2017b). To investigate the anti-apoptosis ability of EGCG-Se NP after SCI, cleaved caspase-3 protein expression was detected at the site injury 24 h after injury. Compared to the saline group, the cleaved caspase-3 level in the EGCG-Se NP group was significantly lower (Supplementary Figure S15). These results suggest that EGCG-Se NP have a significant anti-apoptotic effect after SCI, which may explain the effect of EGCG-Se NP on the recovery of motor function in injured rats.

To comprehensively understand the side effects of EGCG-Se NP treatment, liver and kidney functions were analyzed at days 1 and 56 after injury. There were no significant differences in alkaline phosphatase, alanine aminotransferase, blood urea nitrogen, or serum creatinine levels among all groups, suggesting that EGCG-Se NP treatment causes no side effects to the kidney or liver (Supplementary Figure S16). In addition, the biosafety of EGCG-Se NP was further evaluated by H&E staining of the heart, liver, spleen, lung, and kidney, which showed no significant changes in the EGCG-Se NP and EGCG groups (Supplementary Figure S17).

## Conclusion

In this study, effective neuroprotection after acute SCI was achieved with EGCG-Se NP treatment through ROS scavenging. *In vitro* studies showed that EGCG-Se NP could effectively protect PC12 cells from oxidative stress damage as well as achieve an effective anti-inflammatory effect. Intravenous EGCG-Se NP exert a significant anti-inflammatory and neuroprotective role and effectively promoted functional recovery in a rat model of SCI. Furthermore, this study has demonstrated a safe and prospective pathway to incorporate the merits of selenium and EGCG, thus making it a promising therapeutic for the treatment of SCI or other ROS-mediated conditions.

## Data Availability

The raw data supporting the conclusions of this article will be made available by the authors, without undue reservation.
