# Supplementary material for: Epigallocatechin-3-gallate selenium nanoparticles for neuroprotection by scavenging reactive oxygen species and reducing inflammation
Source: Front Bioeng Biotechnol. 2022 Sep 8;10:989602. doi: 10.3389/fbioe.2022.989602 (PMC9493277; doi:10.3389/fbioe.2022.989602)
Supplement: Supplementary file 1 [file DataSheet1.docx]

**Electronic Supplementary Information**

Epigallocatechin-3-gallate selenium nanoparticles for neuroprotection by scavenging reactive oxygen species and reducing inflammation

Yiming Wang^1^; Wenqi Luo^1^; Feng Lin^1^; Wanguo Liu^1^*; Rui Gu *.

^1^ Department of Orthopaedic Surgery, China-Japan Union Hospital of Jilin University, Changchun 130033, P. R. China

* Correspondence: Wanguo Liu and Rui Gu

Tel: 18186881807 (W. Liu) and 13804371075 (R. Gu);

Fax: 0431-89876921(W. Liu) and 0431-89876939 (R. Gu);

Emails: [liuwanguo6016@jlu.edu.cn](mailto:liuwanguo6016@jlu.edu.cn) (W. Liu) and [gurui@jlu.edu.cn](mailto:gurui@jlu.edu.cn) (R. Gu)


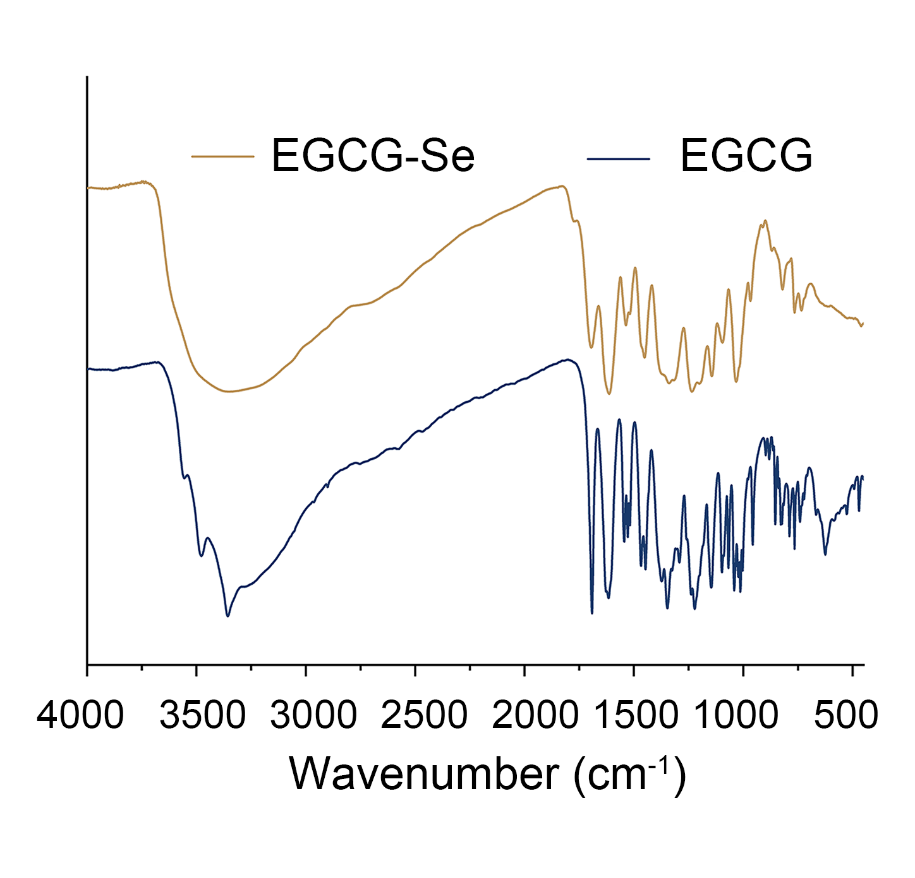


Figure S1. FT-IR of EGCG-Se NPs (nano particals) and EGCG (Epigallocatechin gallate).


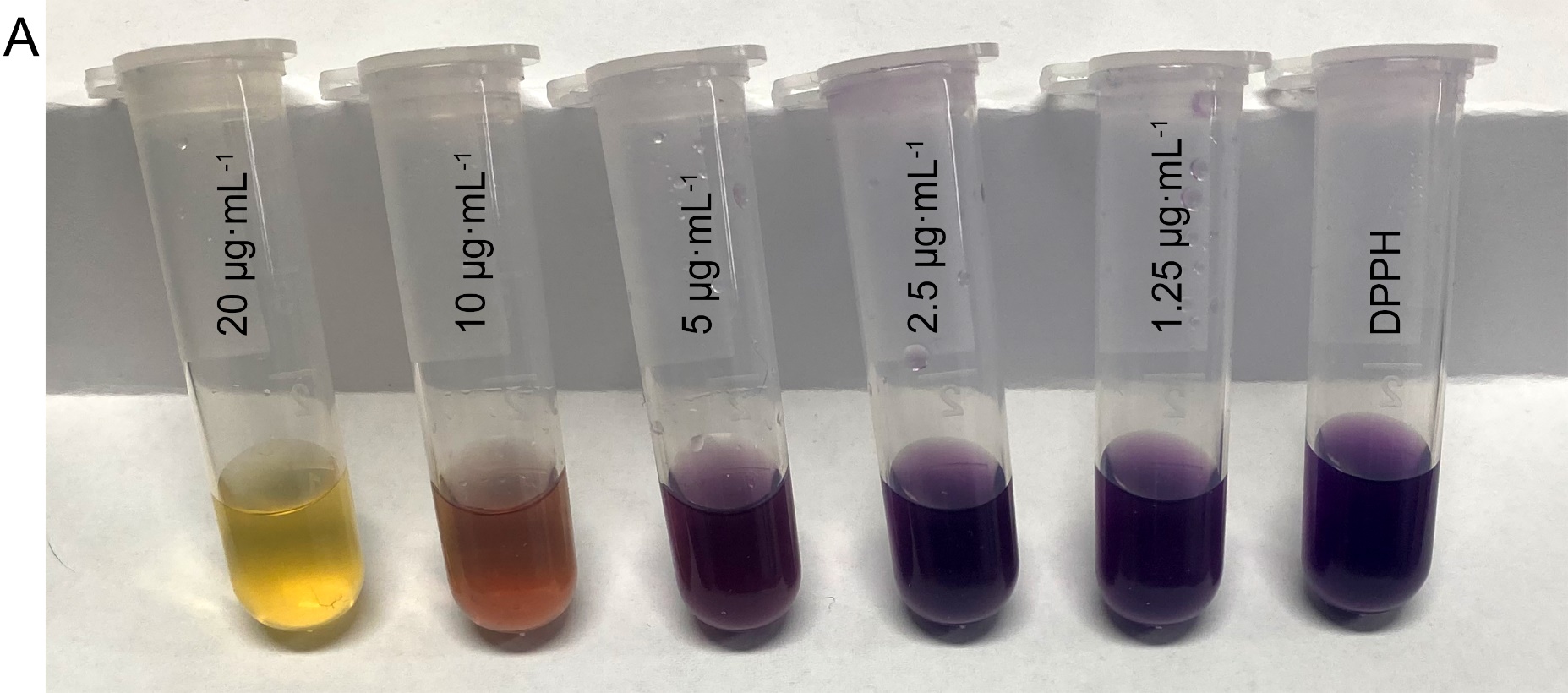


Figure S2. DPPH (1,1-diphenyl-2-picrylhydrazyl) clearance rate of EGCG-Se NP.


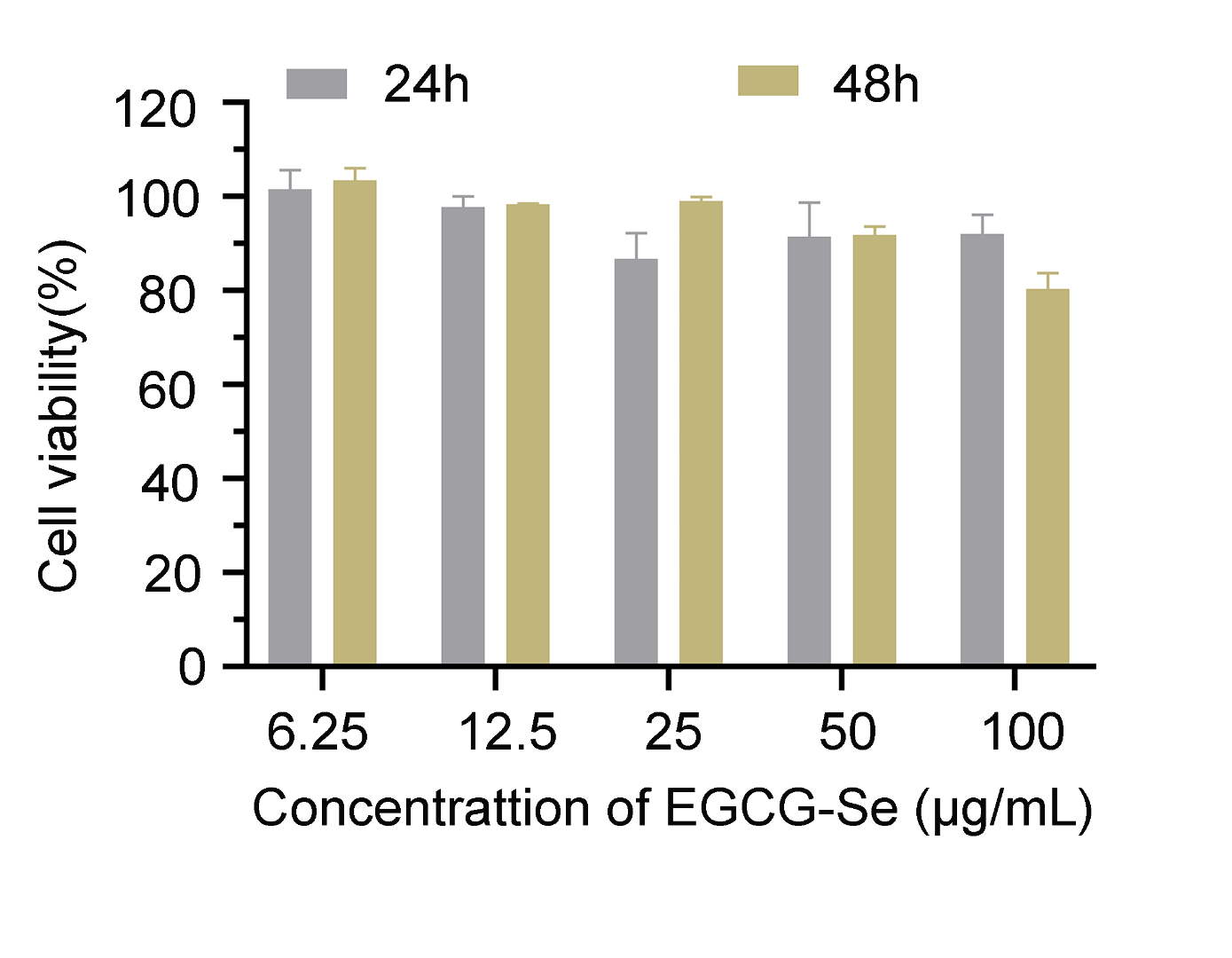


Figure S3. *In vitro* toxicity study of the EGCG-Se NP. Viability of PC12 cells incubated with different concentrations of EGCG-Se NP for 24 h or 48 h. Data are presented as the means ± SDs, with n = 3 for each group.


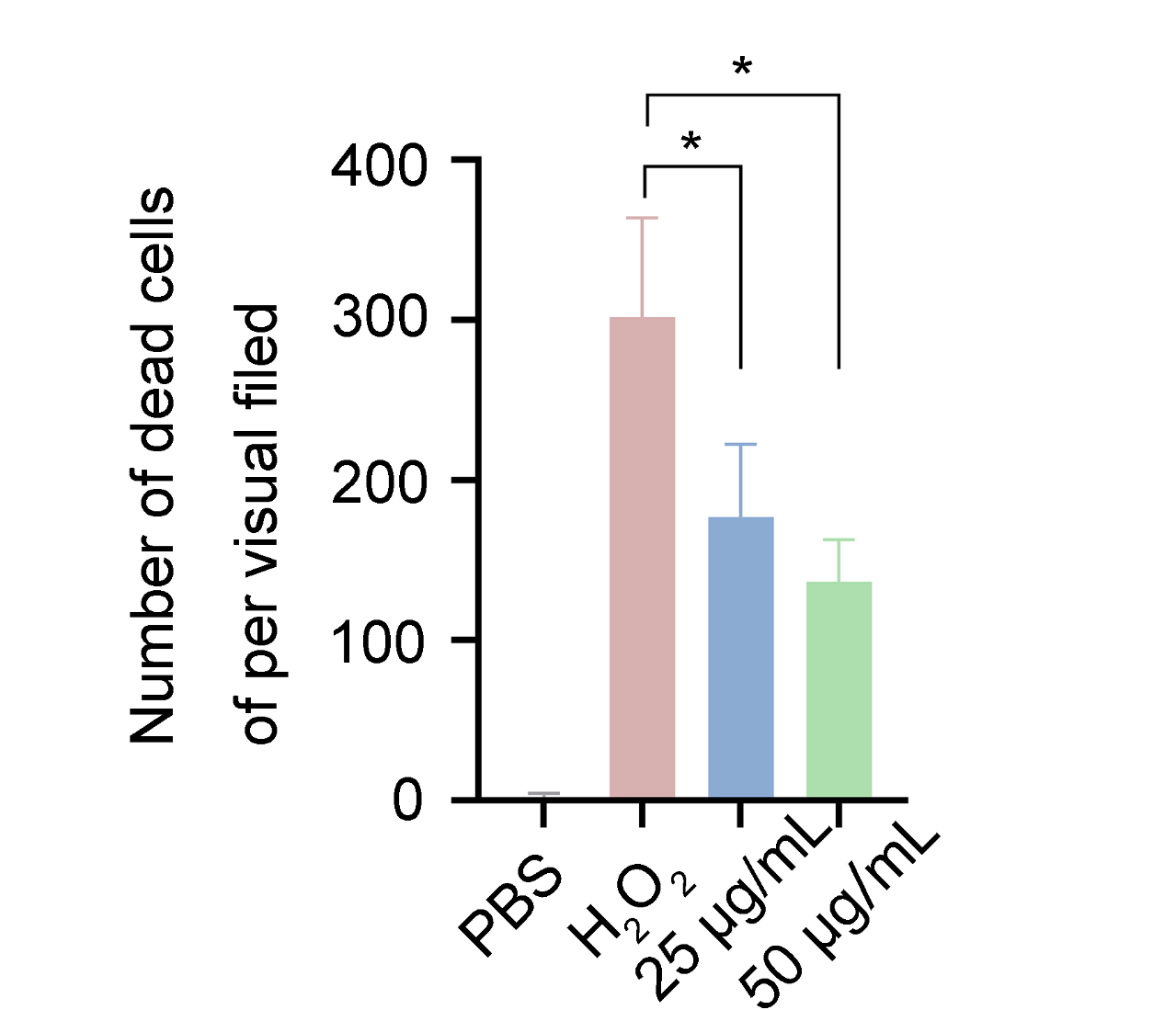


Figure S4. Quantitative analysis of the number of dead cells, * P < 0.05.


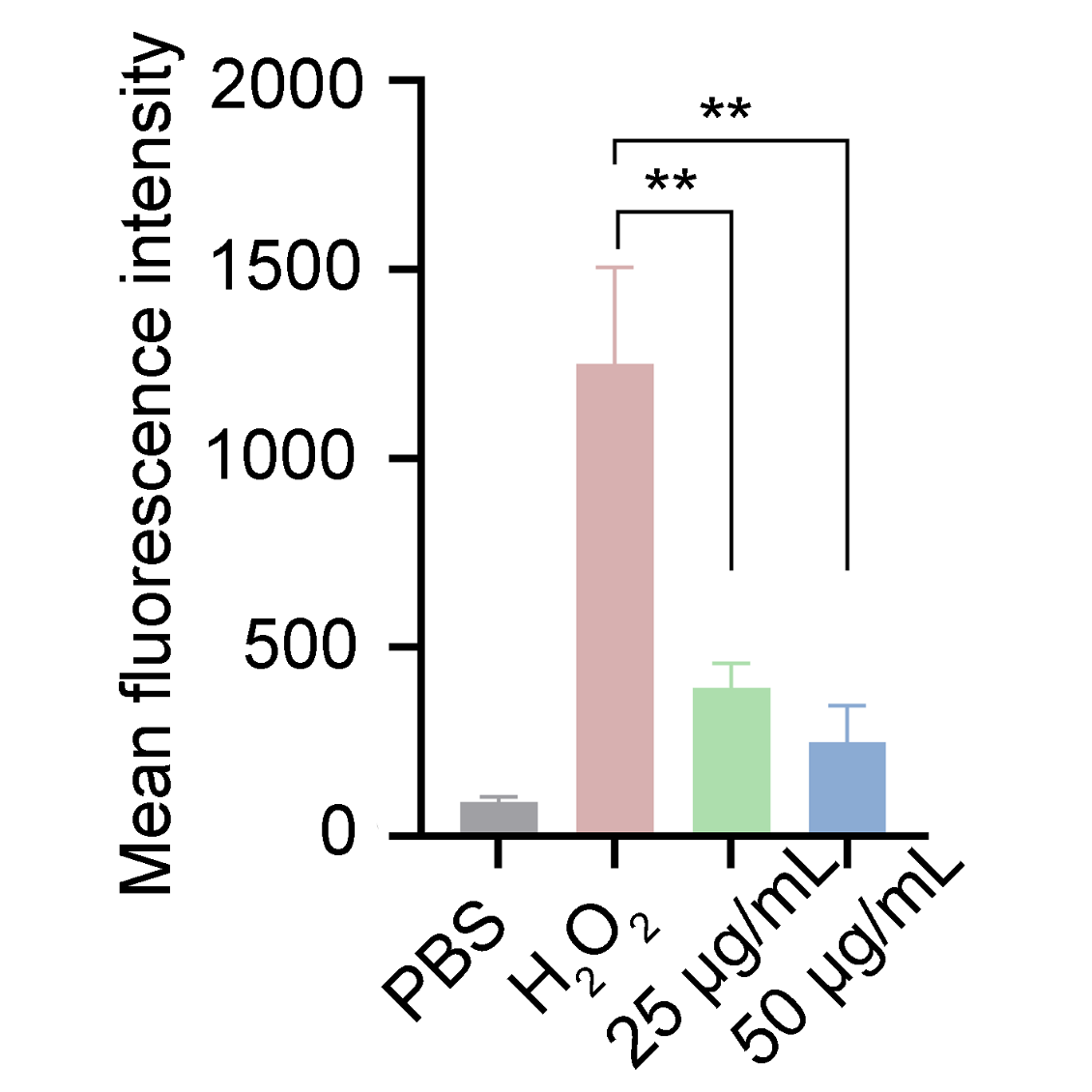


Figure S5. Quantitative analysis of the fluorescence intensity of DCF, ** P < 0.01.


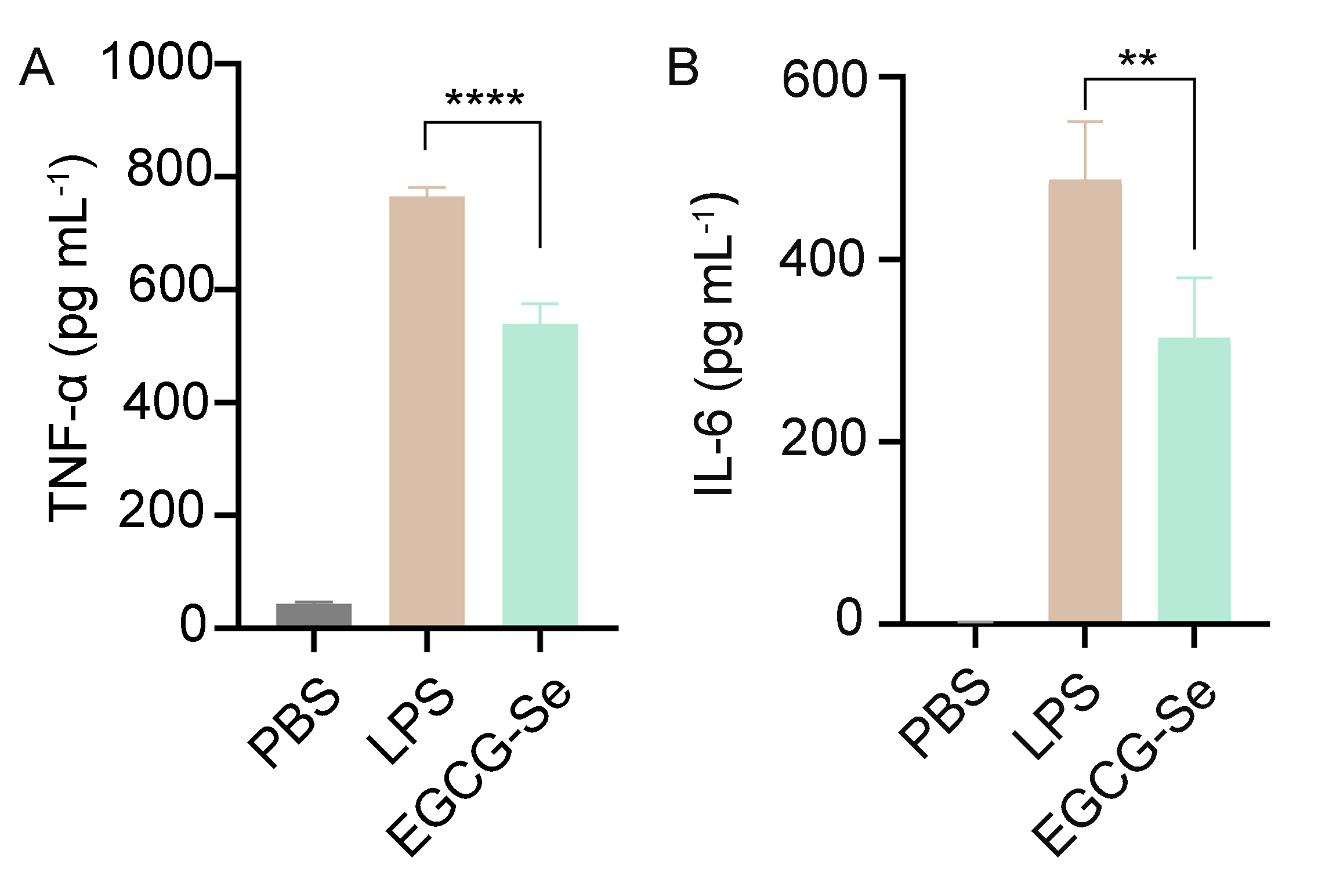


Figure S6. TNF- α and IL-6 levels in the culture medium of LPS-activated microglial cells without or with treatment with 10 μg mL^–1^ EGCG-Se. ∗∗p < 0.01 and ∗∗∗*p < 0.0001.


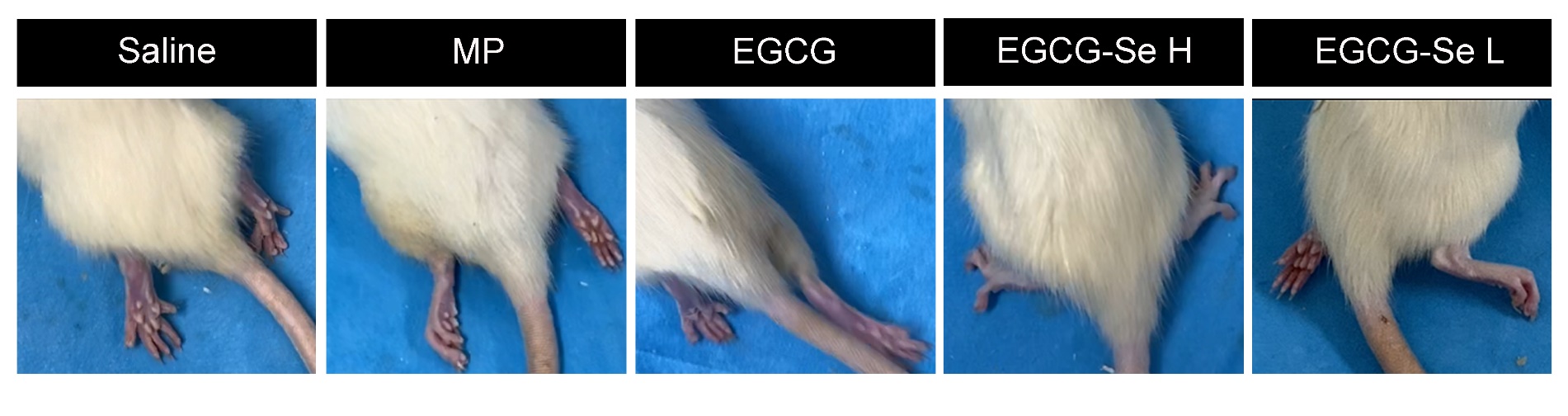


Figure S7. Typical images of the feet in rats with SCI after treatment.


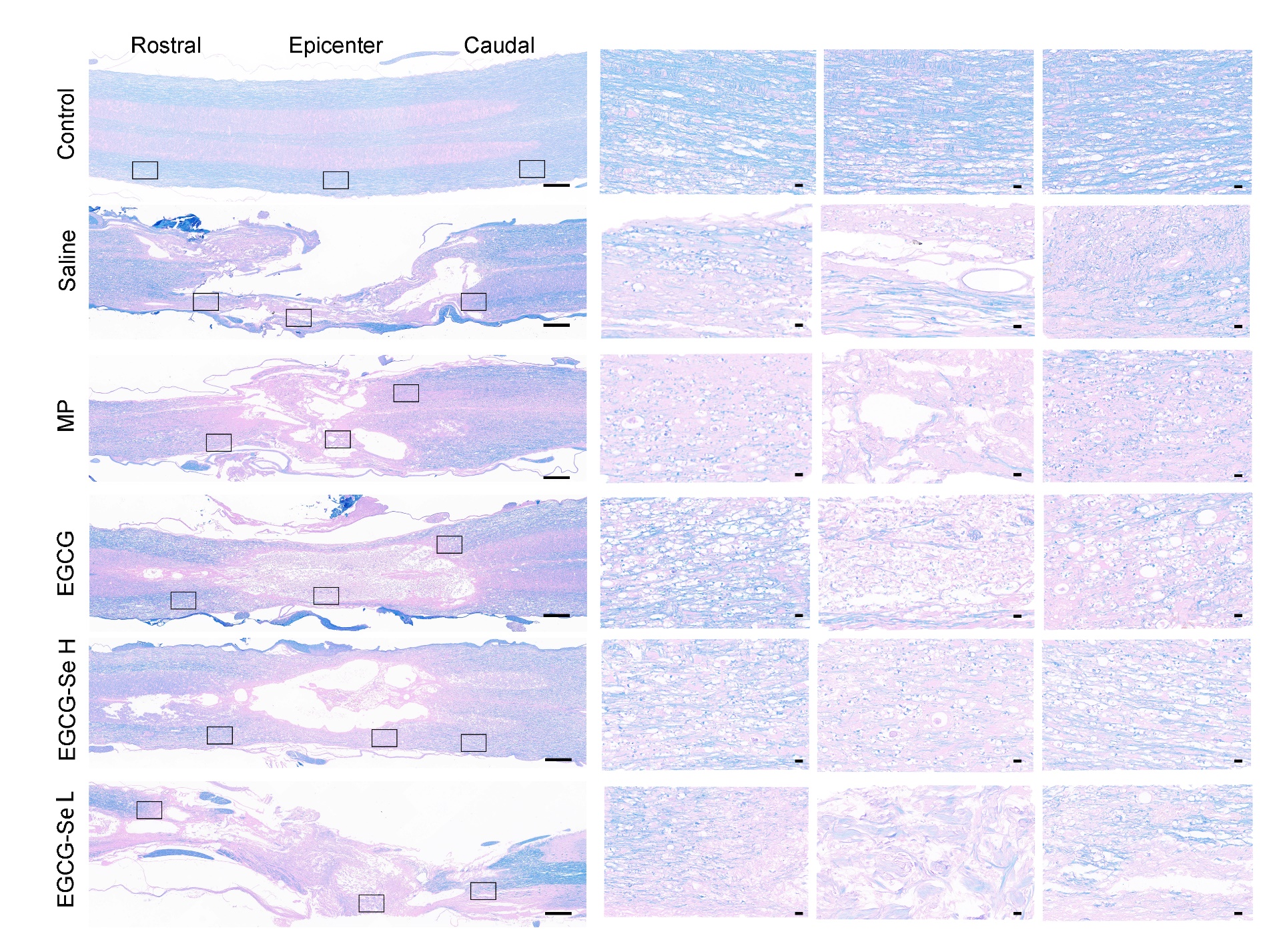


Figure S8. LFB (Luxol Fast Blue) staining of myelin sheaths *in vivo*. LFB staining of myelin sheaths is shown at 2X (left, scale bar = 500 μm) and 20X magnification (right, scale bar = 50 μm).


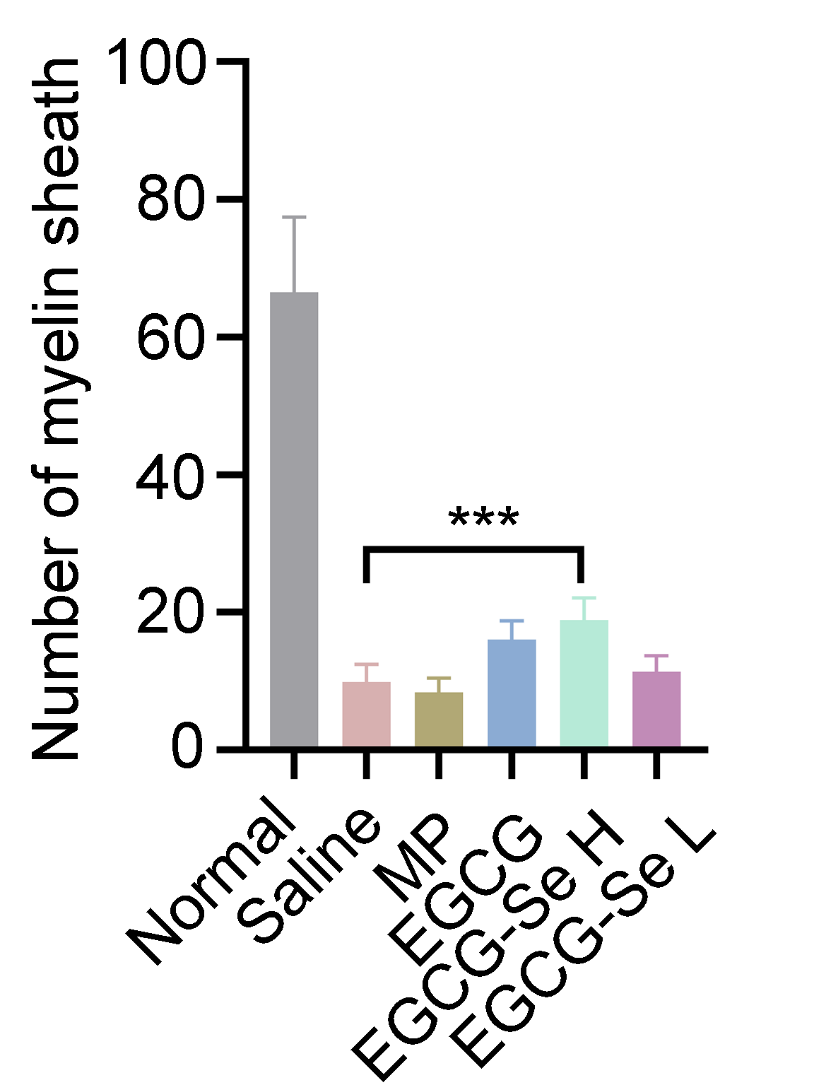


Figure S9. Quantitative analysis of the numbers of myelin sheath in the different groups. ***p < 0.001.


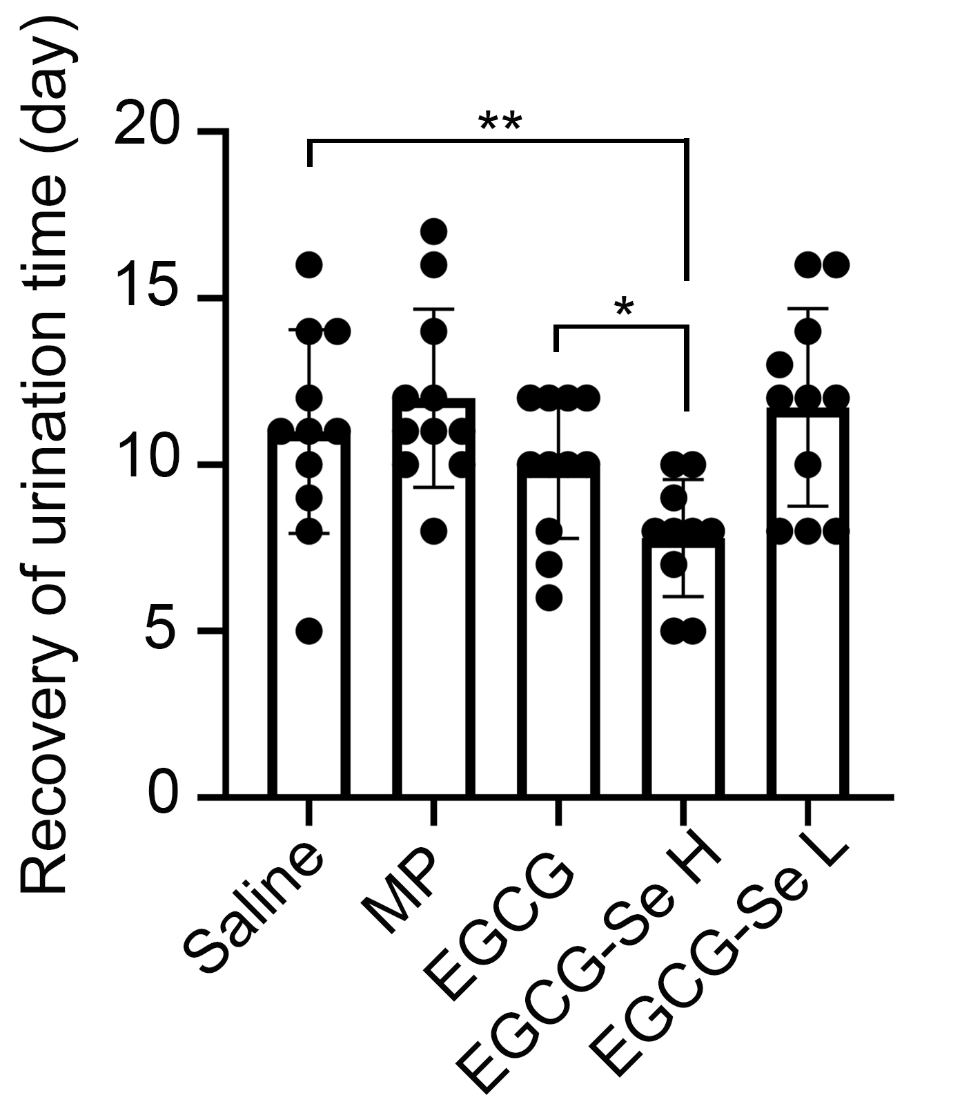


Figure S10. Recovery of the urination time of rats. Recovery of the urination time of rats with SCI after treatment with saline, MP (methylprednisolone), EGCG, 10 mg kg^-1^ EGCG-Se, or 5 mg kg^-1^ EGCG-Se. * P < 0.05, ** P < 0.01.


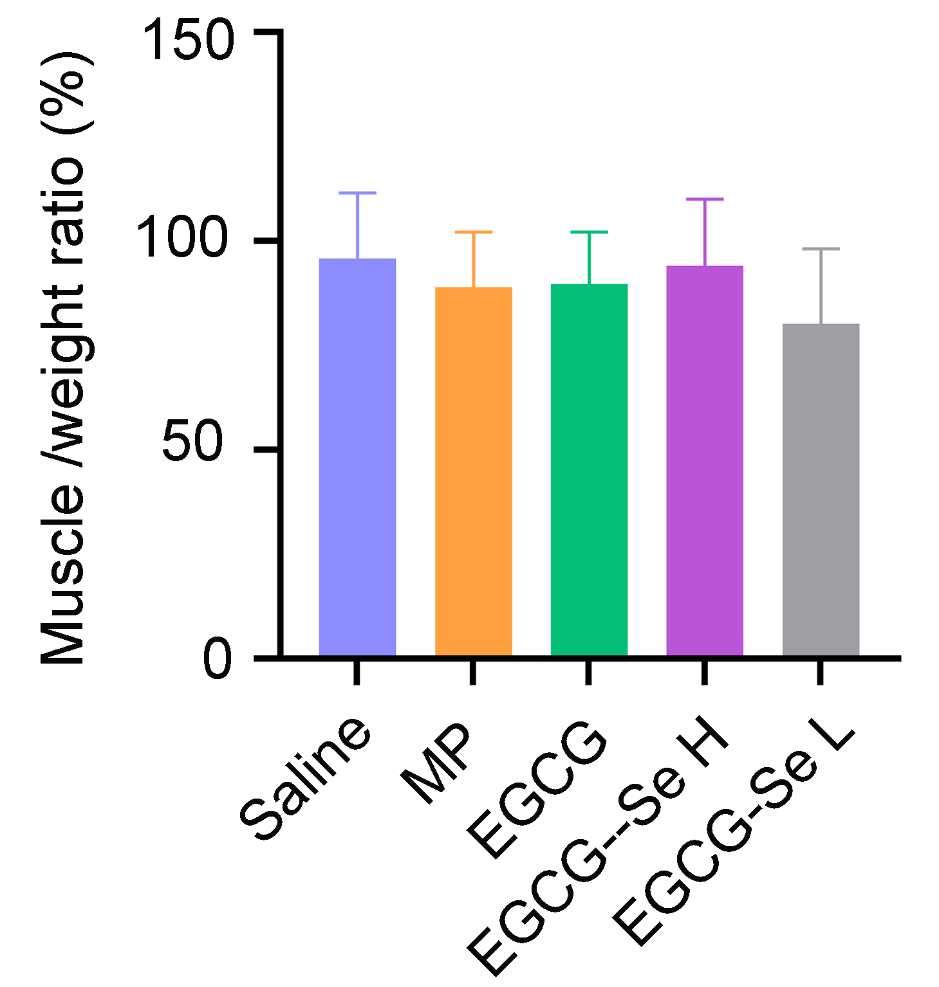


Figure S11. Gastrocnemius muscle/weight ratio.


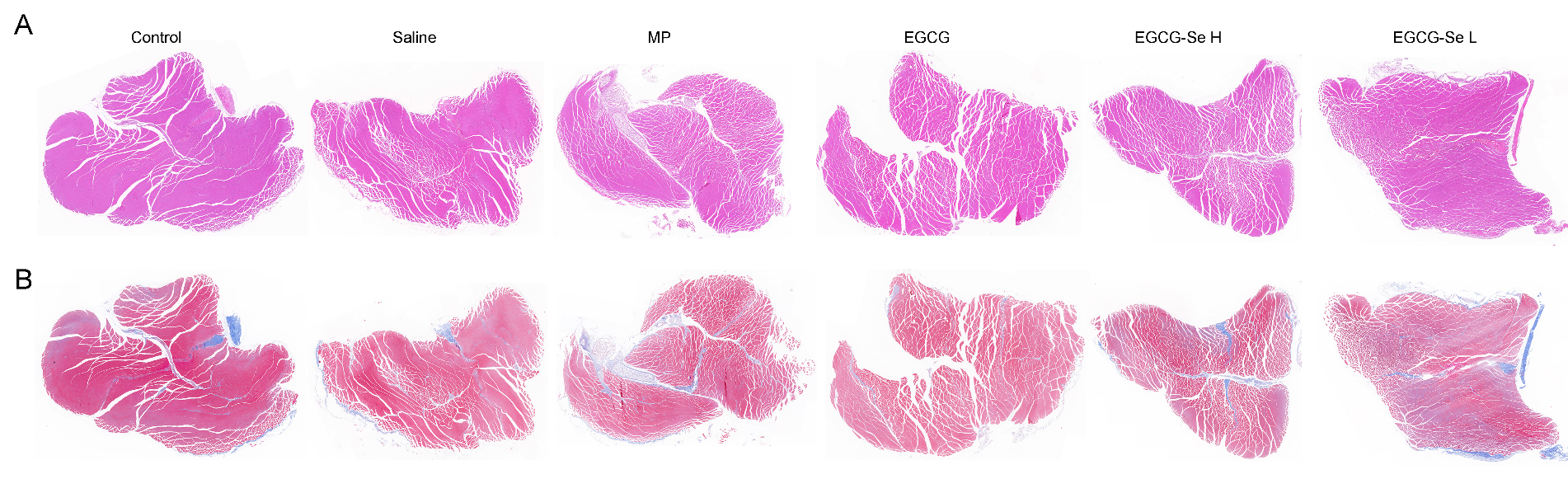


Figure S12. H&E and Masson staining of the gastrocnemius muscle. (A) H&E staining of the gastrocnemius muscle. (B) Masson staining of the gastrocnemius muscle. Scale bar = 500 μm.


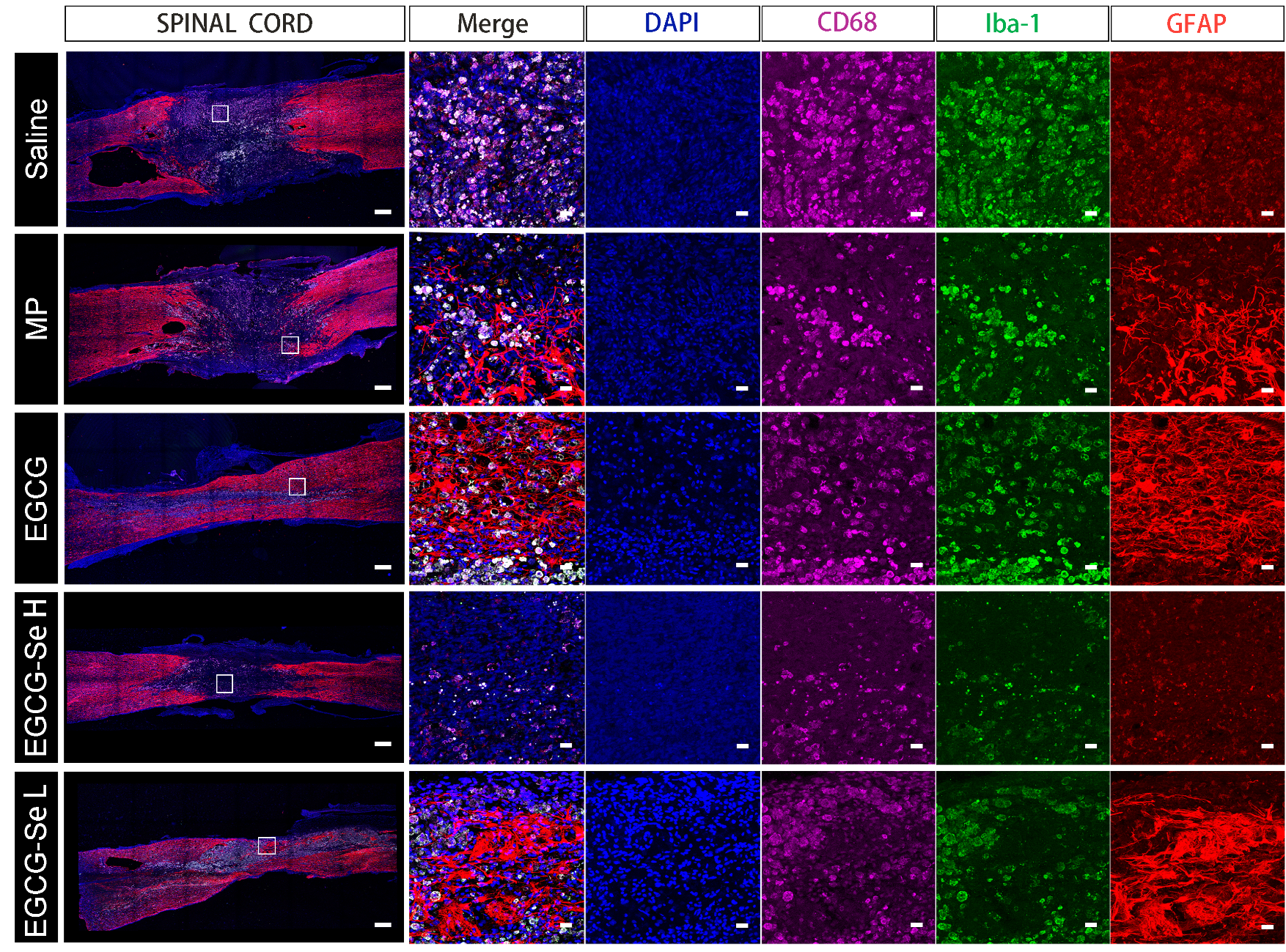


Figure S13 Representative GFAP (red), Iba-1 (green), and CD68 (violet) immunohistochemical images at 8 weeks post injury. Scale bar = 400 µm and 25 μm, respectively.


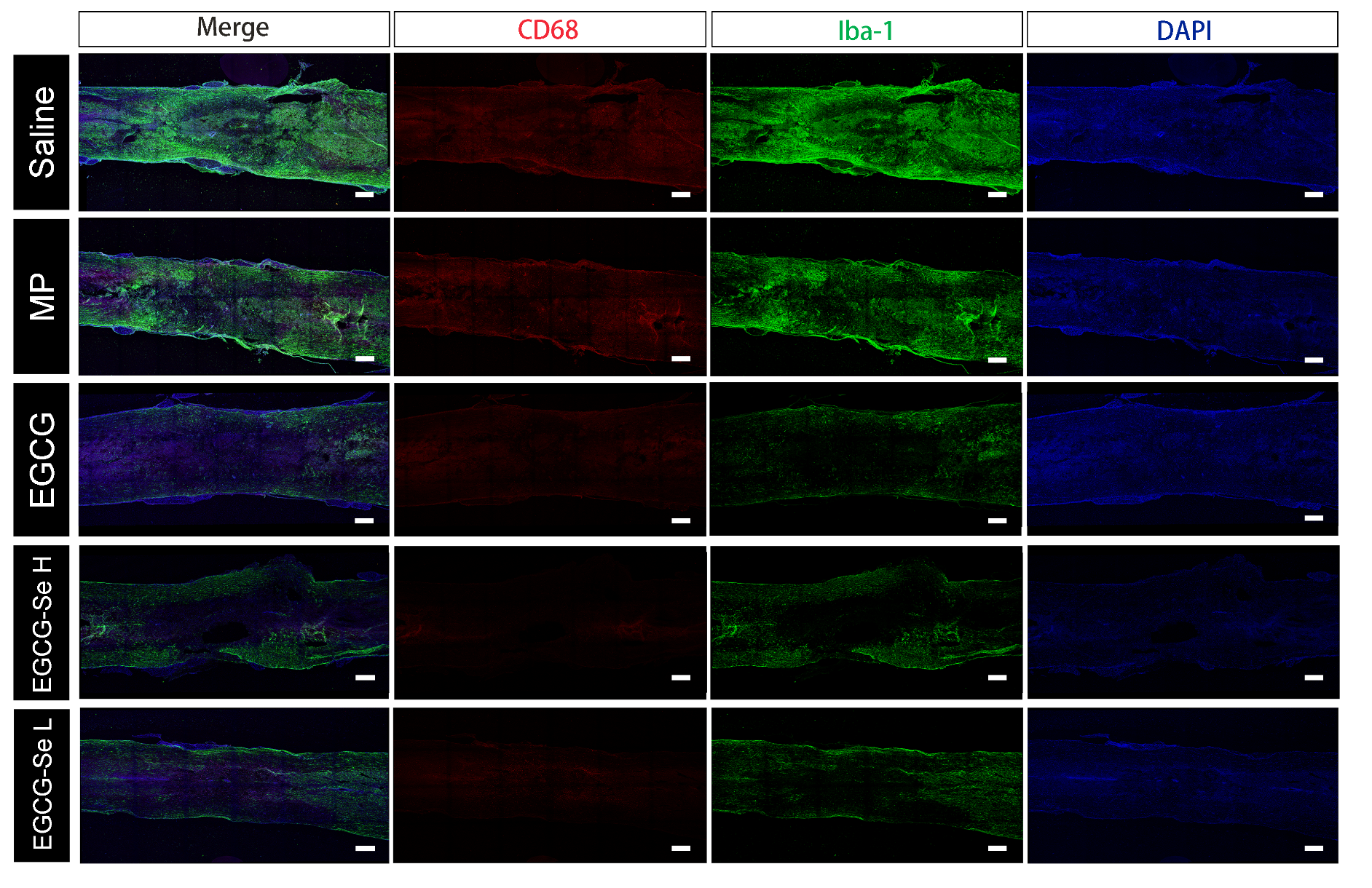


Figure S14. Double-staining of the slices with anti-CD68 and anti Iba-1 antibodies on 1day post operation. Scale bar = 400 μm.


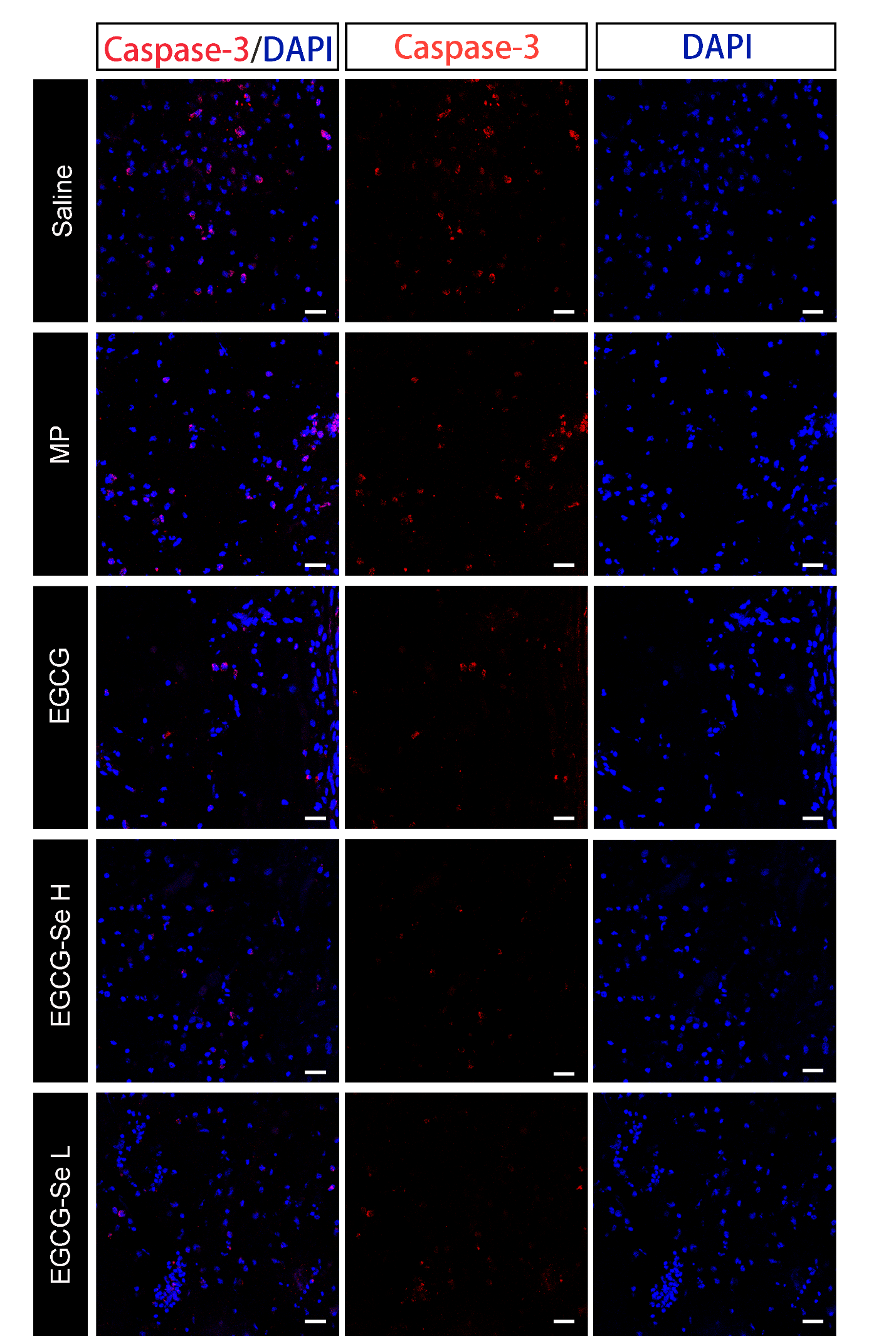


Figure S15. Anti-apoptotic effects of EGCG-Se NPs. Scale bar = 25 μm.


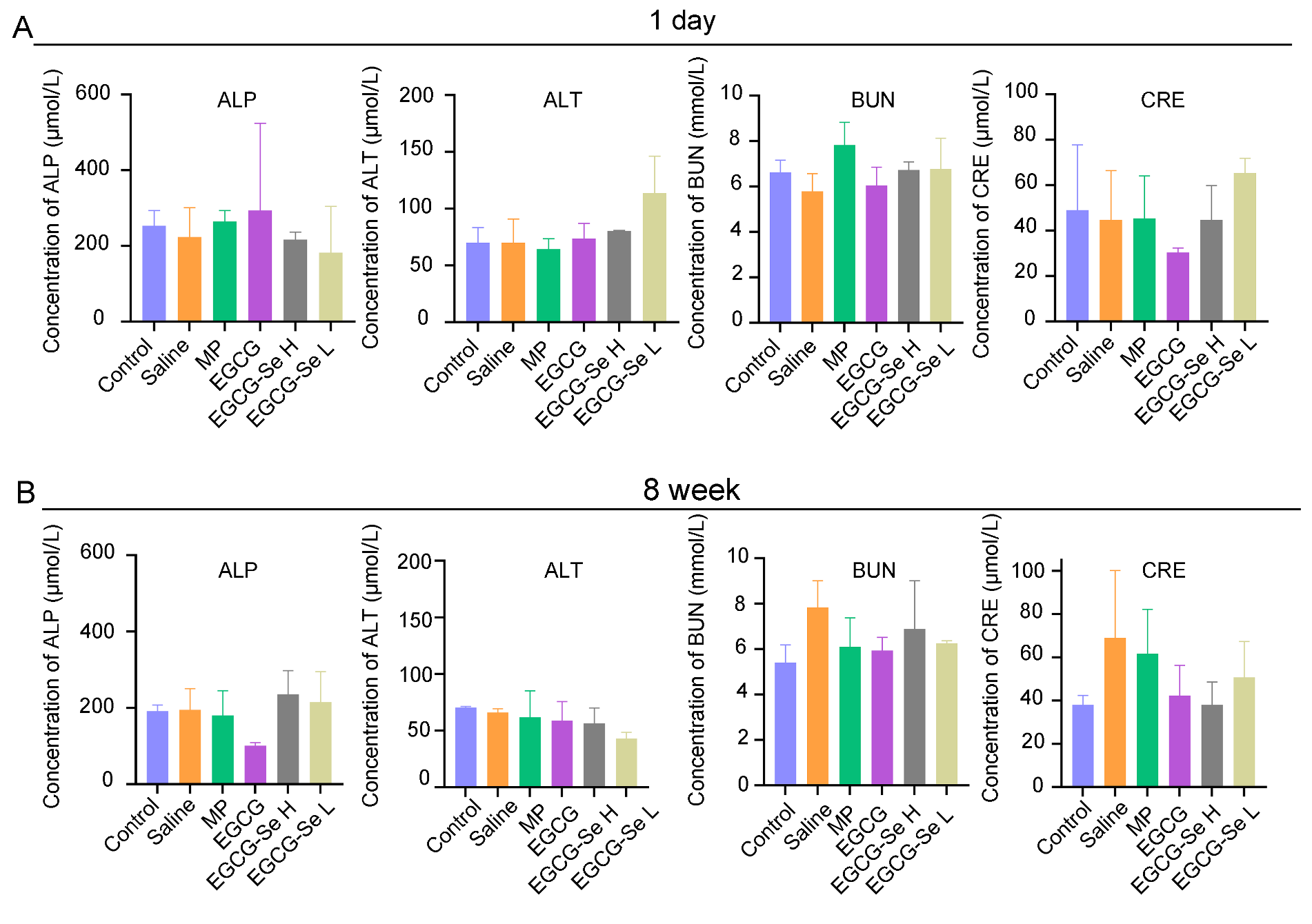


Figure S16. Adverse effects of different treatments on the liver and kidney.ALT (Alanine transaminase),ALP (alkaline phosphatase),BUN (blood urea nitrogen),CRE (Creatinine).


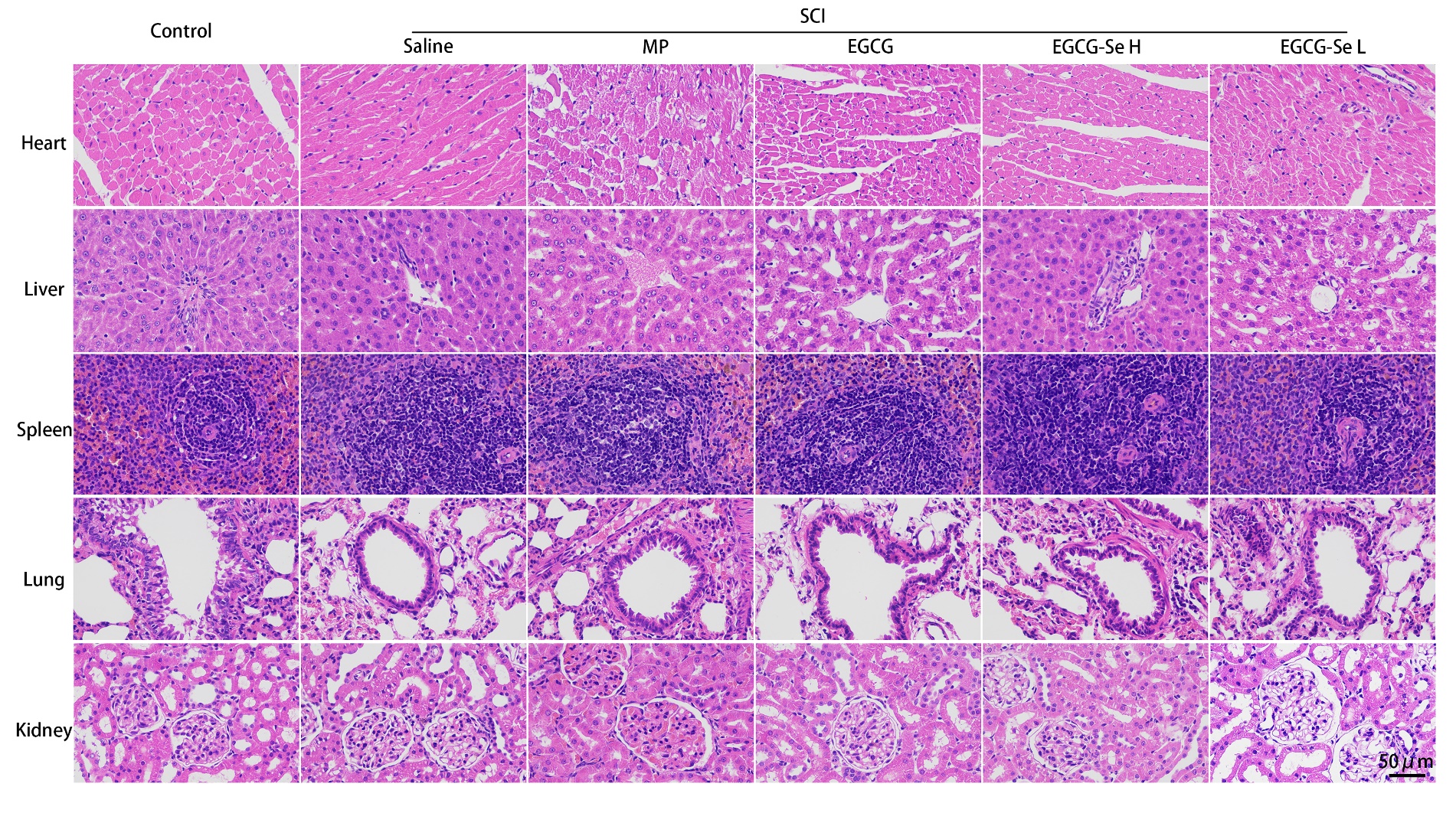


Figure S17. H&E staining of the major organs.
